# Targeting IGF2 to reprogram the tumor microenvironment for enhanced viro-immunotherapy

**DOI:** 10.1093/neuonc/noae105

**Published:** 2024-06-10

**Authors:** Min Hye Noh, Jin Muk Kang, Alexandra A Miller, Grace Nguyen, Minxin Huang, Ji Seon Shim, Alberto J Bueso-Perez, Sara A Murphy, Kimberly A Rivera-Caraballo, Yoshihiro Otani, Eunju Kim, Seung-Hee Yoo, Yuanqing Yan, Yeshavanth Banasavadi-Siddegowda, Hiroshi Nakashima, E Antonio Chiocca, Balveen Kaur, Zhongming Zhao, Tae Jin Lee, Ji Young Yoo

**Affiliations:** Department of Neurosurgery, McGovern Medical School, The University of Texas Health Science Center at Houston, Houston, Texas, USA; Department of Neurosurgery, McGovern Medical School, The University of Texas Health Science Center at Houston, Houston, Texas, USA; Department of Pediatric Hematology & Oncology, University Hospitals Cleveland Medical Center, Cleveland, Ohio, USA; The University of Texas MD Anderson Cancer Center UTHealth Houston Graduate School of Biomedical Science, Houston, Texas, USA; Department of Neurosurgery, McGovern Medical School, The University of Texas Health Science Center at Houston, Houston, Texas, USA; Department of Neurosurgery, McGovern Medical School, The University of Texas Health Science Center at Houston, Houston, Texas, USA; Department of Neurosurgery, McGovern Medical School, The University of Texas Health Science Center at Houston, Houston, Texas, USA; Department of Neurosurgery, McGovern Medical School, The University of Texas Health Science Center at Houston, Houston, Texas, USA; Department of Neurosurgery, McGovern Medical School, The University of Texas Health Science Center at Houston, Houston, Texas, USA; The University of Texas MD Anderson Cancer Center UTHealth Houston Graduate School of Biomedical Science, Houston, Texas, USA; Georgia Cancer Center and Department of Pathology, Medical College of Georgia, Augusta University, Augusta, Georgia, USA; The University of Texas MD Anderson Cancer Center UTHealth Houston Graduate School of Biomedical Science, Houston, Texas, USA; Georgia Cancer Center and Department of Pathology, Medical College of Georgia, Augusta University, Augusta, Georgia, USA; Department of Neurological Surgery, Okayama University Graduate School of Medicine, Dentistry, and Pharmaceutical Sciences, Okayama, Japan; Department of Neurosurgery, McGovern Medical School, The University of Texas Health Science Center at Houston, Houston, Texas, USA; Department of Food and Nutriton, Kongju National University, Yesan, Chungnam, South Korea; Department of Biochemistry, McGovern Medical School, The University of Texas Health Science Center at Houston, Houston, Texas, USA; Department of Biochemistry, McGovern Medical School, The University of Texas Health Science Center at Houston, Houston, Texas, USA; Department of Surgery, Northwestern University Feinberg School of Medicine, Chicago, Illinois, USA; Department of Neurosurgery, McGovern Medical School, The University of Texas Health Science Center at Houston, Houston, Texas, USA; Surgical Neurology Branch, National Institute of Neurological Disorders and Stroke, National Institutes of Health, Bethesda, Maryland, USA; Department of Neurosurgery, Brigham and Women’s Hospital and Harvard Medical School, Boston, Maryland, USA; Department of Neurosurgery, Brigham and Women’s Hospital and Harvard Medical School, Boston, Maryland, USA; Georgia Cancer Center and Department of Pathology, Medical College of Georgia, Augusta University, Augusta, Georgia, USA; Center for Precision Health, McWilliams School of Biomedical Informatics, The University of Texas Health Science Center at Houston, Houston, Texas, USA; The University of Texas MD Anderson Cancer Center UTHealth Houston Graduate School of Biomedical Science, Houston, Texas, USA; Department of Neurosurgery, McGovern Medical School, The University of Texas Health Science Center at Houston, Houston, Texas, USA; The University of Texas MD Anderson Cancer Center UTHealth Houston Graduate School of Biomedical Science, Houston, Texas, USA; Department of Neurosurgery, McGovern Medical School, The University of Texas Health Science Center at Houston, Houston, Texas, USA

**Keywords:** glioblastoma (GBM), insulin-like growth factor 2 (IGF2), insulin-like growth factor-1 receptor (IGF1R), Oncolytic herpes simplex virus-1 (oHSV), tumor microenvironment (TME)

## Abstract

**Background:**

The FDA approval of oncolytic herpes simplex-1 virus (oHSV) therapy underscores its therapeutic promise and safety as a cancer immunotherapy. Despite this promise, the current efficacy of oHSV is significantly limited to a small subset of patients largely due to the resistance in tumor and tumor microenvironment (TME).

**Methods:**

RNA sequencing (RNA-Seq) was used to identify molecular targets of oHSV resistance. Intracranial human and murine glioma or breast cancer brain metastasis (BCBM) tumor-bearing mouse models were employed to elucidate the mechanism underlying oHSV therapy-induced resistance.

**Results:**

Transcriptome analysis identified IGF2 as one of the top-secreted proteins following oHSV treatment. Moreover, IGF2 expression was significantly upregulated in 10 out of 14 recurrent GBM patients after treatment with oHSV, rQNestin34.5v.2 (71.4%; *P* = .0020) (ClinicalTrials.gov, NCT03152318). Depletion of IGF2 substantially enhanced oHSV-mediated tumor cell killing in vitro and improved survival of mice bearing BCBM tumors in vivo. To mitigate the oHSV-induced IGF2 in the TME, we constructed a novel oHSV, oHSV-D11mt, secreting a modified IGF2R domain 11 (IGF2RD11mt) that acts as IGF2 decoy receptor. Selective blocking of IGF2 by IGF2RD11mt significantly increased cytotoxicity, reduced oHSV-induced neutrophils/PMN-MDSCs infiltration, and reduced secretion of immune suppressive/proangiogenic cytokines, while increased CD8 + cytotoxic T lymphocytes (CTLs) infiltration, leading to enhanced survival in GBM or BCBM tumor-bearing mice.

**Conclusions:**

This is the first study reporting that oHSV-induced secreted IGF2 exerts a critical role in resistance to oHSV therapy, which can be overcome by oHSV-D11mt as a promising therapeutic advance for enhanced viro-immunotherapy.

## Graphical Abstract

**Graphical Abstract:**
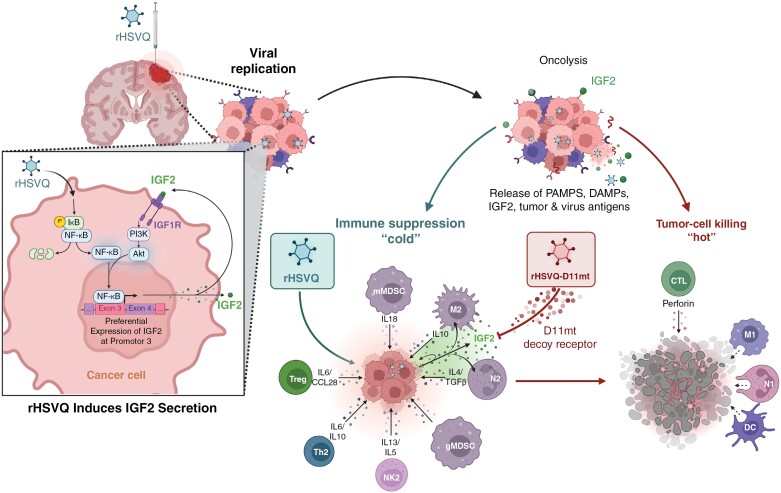

**Key Points**

1. oHSV therapies induce IGF2 expression and secretion into the TME, hampering therapeutic efficacy.
2. Targeted inhibition of IGF2 reshapes the TME, enhancing the therapeutic efficacy of viro-immunotherapy.

**Importance of the Study**

Herpes simplex virus-1-derived oncolytic virus (oHSV) is the most advanced virotherapy as approved by the FDA for melanoma in the United States and conditionally for recurrent glioblastoma patients in Japan. While oHSV therapy has demonstrated therapeutic promise against several types of cancer including GBM, only a select group of patients experience robust and long-term responses in the clinic. Thus, elucidating the mechanisms by which cancer cells develop resistance to oHSV therapy is essential in maximizing patient outcomes. In this study, we discovered that oHSV therapy-induced expression and secretion of Insulin-like Growth Factor 2 (IGF2) is a critical driver in developing resistance to oncolytic viro-immunotherapy. Mitigating IGF2 within the TME utilizing oHSV-D11mt, a novel next-generation oHSV, reprograms the tumor microenvironment (TME), enhancing viro-immunotherapy. Finally, our study provides a novel paradigm for overcoming the resistance to viro-immunotherapy.

Oncolytic herpes simplex virus-1 (oHSV) therapy is the only oncolytic virus (OV) approved by the FDA in the United States for use in patients with metastatic melanoma.^1^ OVs work both through direct oncolysis of infected cancer cells and induction of antitumor immunity through the release of tumor antigens from the lysed cancer cells, a phenomenon referred to as viro-immunotherapy. Both preclinical and clinical data suggest that OV therapy-induced oncolysis and antiviral immune responses can remodel a “cold” tumor microenvironment (TME) with few immune effector cells to a “hot” environment with increased infiltration of tumor-reactive lymphocytes. However, only a limited subset of patients generate a robust long-term response.^2,3^ Thus, elucidating the tumor-TME interplay in the context of viro-immunotherapy will uncover unique vulnerabilities that can be exploited to augment therapeutic outcomes.

The insulin-like growth factor (IGF) system is a highly conserved signaling pathway implicated in numerous malignancies and is composed of ligands (IGF1 and IGF2), receptors (IGF1R and IGF2R), and high-affinity IGF binding proteins (IGFBP1 to IGFBP6).^4^ Upon binding to its ligands, IGF1R activates the downstream signaling pathways (eg, PI3K/AKT and MAPK/ERK), increasing cell proliferation, migration, invasion, and survival. Importantly, overexpression of IGF2 and the resultant activation of the IGF1R signaling pathway has been shown to be significantly associated with poor survival in GBM and breast cancer (BC) patients.^5–8^ Furthermore, several recent studies have shown that IGF2 secreted by immunosuppressive TME cells (eg, tumor-associated macrophages (TAMs), cancer-associated fibroblasts, and endothelial cells) promotes an immunosuppressive TME, advancing tumor progression and making traditional immunotherapies largely unsuccessful.^9–12^ Thus, strategies to therapeutically modulate IGF1R signaling are of great interest in the treatment of GBM and BC brain metastasis (BCBM). However, systemic IGF1R inhibitors such as monoclonal IGF/IGF1R antibodies and small molecule inhibitors (eg, OSI-906) have yet to show meaningful outcomes in the clinic.^13,14^

In this study, we discovered a novel mechanism of resistance to oHSV therapy in which viral therapy-induced IGF2 expression and secretion activates IGF2/IGF1R signaling in tumor and TME cells. This led to tumor regrowth and promoted evasion of antitumor immunity, ultimately hindering therapeutic efficacy. To mitigate this resistance, we developed a novel next-generation oHSV, oHSV-D11mt, which expresses a secretable IGF2R domain 11 that functions as an IGF2 decoy receptor. Further, we demonstrate that oHSV-D11mt infection specifically neutralized IGF2 not IGF1 within the TME, effectively abrogating the resistance conferred by IGF2/IGF1R signaling in both tumor and neutrophils/polymorphonuclear myeloid-derived suppressor cells (PMN-MDSCs). Importantly, IGF2 inhibition impeded the formation of an immunosuppressive TME and enhanced the recruitment of antitumor CD8 + cytotoxic T-lymphocytes (CTLs), resulting in markedly improved survival of mice bearing GBM and BCBM tumor. Furthermore, oHSV-D11mt sensitized tumors to adjuvant immune checkpoint blockade (ICB) (i.e. anti-PD-L1 therapy). Ultimately, innovative approaches to maximizing antitumor immunity generated by oHSV treatment are critical to augmenting their success in the clinic and ensuring maximum therapeutic efficacy in a highly diverse patient population.

## Materials and Methods

### Ethics Statement

All mouse housing and experiments were performed in accordance with the guidelines set by the Animal Welfare Committee at the University of Texas Health Science Center in Houston and have been approved by the Institutional Review Board. The clinical trial was performed under IND 016380 and registered as NCT03152318. The clinical trial was approved by the IRB of Dana Farber Cancer Institute/ Brigham and Women’s Hospital.

### Cell lines and Oncolytic Herpes Simples Virus-1 (oHSV-1)

All cell lines, primary GBM cells, and viruses used in this study are described in Supplementary Materials and Methods sections.

### RNA Sequencing (RNA-Seq) and Data Analysis

RNA-seq was performed for GBM12 and MDA468 cells infected with or without 0.1 MOI of rHSVQ. Sixteen hours post viral infection, total RNA was isolated and sequenced. Details of this analysis are described in Supplementary Materials and Methods.

### Dual Luciferase-NFκB Promoter Assay, Chromatin Immunoprecipitation (ChIP) Assays, qRT-PCR, Quantification of IGF2 ELISA, Cell Proliferation Assay, Binding Affinity Assay, Western Blotting, Immunohistochemistry, and Flow Cytometry.

All commercial kits, primers, and antibodies used in these experiments are listed in Supplementary Table S1-S3. Detailed descriptions of these methods can be found in Supplementary Meterials and Methods.

### Animal Studies

Six- to eight-week-old outbred male and female athymic nu/nu, NSG, C57BL/6, BALB/C, and FVB/N mice were purchased from Jackson Laboratory (Bar Harbour, ME, USA). All details of orthotropic intracranial tumor implantation and treatment are described in Supplementary Materials and Methods.

### Statistical Analysis

Statistical analyses were performed using GraphPad Prism version 10 (GraphPad, San Diego, CA). Student’s *t*-test or Mann–Whitney U test was used to test the difference in comparison of continuous data between the 2 groups. To analyze survival data, Kaplan–Meier curves were compared using the log-rank test and the post hoc pairwise groups test (if applicable) was further performed by Benjamini and Hochberg correction. A *P* value less than .05 was considered statistically significant.

## Results

### oHSV Treatment Significantly Induces Secretion of Insulin-Like Growth Factor 2 (IGF2) in Preclinical and Clinical Models of GBM and BCBM

**Figure 1.**
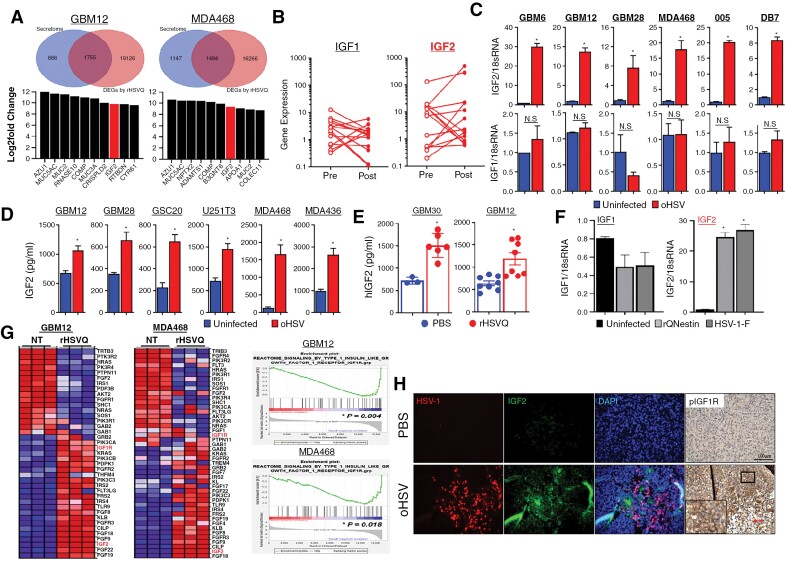
oHSV therapy induces IGF2 gene expression in virus-infected tumor cells. (A) mRNA-Seq of patient-derived primary GBM (GBM12) and MDA468 human BC cells (*n* = 4/group) treated with or without rHSVQ (MOI = 0.1) for 16 hours. The Venn diagram depicts the number of differentially expressed genes (DEGs) (top) and a list of top 10 most upregulated secretome genes following rHSVQ infection (bottom) in GBM12 and MDA468 cells. (B) IGF1 and IGF2 gene expression levels pre- and post-rQNestin34.5v2 treatment in 14 recurrent GBM patients (ClinicalTrials.gov, NCT03152318). (C-D) Validation of IGF2 and IGF1 gene expression and proten secretion by quantitative RT-PCR (qRT-PCR) and ELISA in vitro. Various human primary GBM and U251T3 glioma and breast cancer (BC) cell lines were infected with or without rHSVQ (MOI = 0.1 ~ 1 MOI). Twenty-four hours post-viral infection, cells and culture media (CM) were collected for qRT-PCR (C) and human IGF2 ELISA (D), respectively. (E) Quantification of secreted IGF2 levels in intracranial GBM tumors (GBM12 and GBM30) treated with PBS or oHSV in vivo. Ten days post-tumor implantation, tumor-bearing brain hemispheres were injected intratumorally with PBS or rHSVQ (5 × 10^5^ pfu). Tumor-bearing brain hemispheres were collected 1 day post-treatment and homogenized in serum-free DMEM media (GBM30: PBS, *n* = 3; rHSVQ, *n* = 6 and GBM12: *n* = 8/group). Data shown are the mean ± SEM. *P < .05, NS = not significant. (F) qRT- PCR of IGF2 gene expression in GBM12 cells infected with various HSV-1 viruses (rQNestin34.5v.1 and wild-type F strain) (MOI = 0.5). GBM12 cells were infected with various HSV-1 for 24 hours. IGF1 and IGF2 gene expression was measured with qRT-PCR, using 18S rRNA as an expression normalization control. Data shown are mean fold-change in gene expression ± S.D., normalized to uninfected cells (*n* = 3/group). *P < .05. (G) Expression heatmap and (left) GSEA plots (right) of IGF1R signaling in GBM12 and MDA468 cells from (A). (H) Histological analysis of rHSVQ treatment-induced IGF2-IGF1R signaling activation in intracranial GBM12 tumor-bearing brain tissue sections from mice treated with PBS or rHSVQ. Representative fluorescent microscopy images staining for HSV-1 (red), IGF2 (green), DAPI (blue), and pIGF1R (DAB). (Magnification, 4X).

To screen for mechanisms of resistance to oHSV therapy, we performed global transcriptomic analysis by mRNA sequencing (mRNA-Seq) of patient-derived primary GBM (GBM12) and BC (MDA468) cells treated with rHSVQ, which is an F-strain HSV-1 carrying a double deletion of the neurovirulence factor γ34.5 and viral ribonucleotide reductase ICP6 for 16 hours.^15^ The mRNA-seq identified 7078 and 6843 differentially expressed genes that were upregulated and 13 916 and 10 980 that were down-regulated after rHSVQ infection in GBM12 and MDA468 cells, respectively (Supplementary Figure 1A). Next, we examined the secretome, finding 1755 differentially expressed genes in GBM12 and 1494 in MDA468 that were significantly altered following rHSVQ infection (Figure 1A, FC > 1.5, FDR < 0.05). We identified commonly upregulated secretome genes in both GBM12 and MDA468, implicating previously undescribed genes including AZU1, MUC5AC, COMP, MUC2, and IGF2 (Figure 1A). Interestingly, among the fourteen recurrent GBM patients treated with rQNestin34.5v.2 (ClinicalTrials.gov, NCT03152318), the IGF2 expression level was significantly increased in ten patients out of fourteen (71.4%) after rQNestin34.5v.2 treatment (*P*  = .0020).^16^ However, the expression level of the IGF1 gene was increased in only four patients (28.6%; *P*  = .1250; Figure 1B). A similar observation was reported in a recent phase 1b clinical trial of G207, an rHSVQ-like oHSV (ClinicalTrials.gov, NCT03911388 and NCT02457845).^17^

To validate IGF2 gene induction by rHSVQ infection in our preclinical models, we performed qRT-PCR analysis of various primary GBM, BC, and murine glioma cells following rHSVQ infection. IGF2 expression was significantly upregulated after rHSVQ infection in all cells tested, showing up to a 30-fold increase in human GBM (GBM6) and a 20-fold increase in murine GBM (005; Figure 1C, top). Furthermore, the rHSVQ-induced IGF2 gene expression was MOI- and time-dependent (Supplementary Figure 1C). Similar to our clinical findings, the mRNA level of IGF1 was not significantly affected (Figure 1C, bottom). Moreover, IGF2 secretion was significantly increased upon rHSVQ infection while IGF1 was unaffected as confirmed by ELISA (Figure 1D; Supplementary Figure 1D). A significant increase in IGF2 expression was further confirmed in vivo by ELISA of tumor lysates of intracranial GBM12 or GBM30 tumor-bearing mice treated with rHSVQ when compared to PBS control (Figure 1E). To determine whether IGF2 up-regulation was specific to rHSVQ, GBM12 cells were infected with various types of HSV-1 (eg, wild-type F-strain HSV-1 and rQNestin34.5v.1) and the mRNA expression level of IGF2 was determined by qRT-PCR. Both wild-type HSV and rQNestin34.5v.1 significantly increased the expression of IGF2, but not IGF1 (Figure 1F), indicating that up-regulation of IGF ligands by oHSV infection is limited to IGF2 regardless of the virus type.

Gene set enrichment analysis on the mRNA-seq data obtained in Figure 1A revealed that the genes in the IGF2-IGF1R pathway were significantly enriched in both GBM12 and MDA468 cells after rHSVQ infection (Figure 1G). Similarly, histological analysis of brain sections obtained from GBM12 tumor-bearing mice demonstrated a significant increase in IGF2 expression and IGF1R phosphorylation co-localized within the region of active rHSVQ replication (Figure 1H). Interestingly, Chinese Glioma Genome Atlas (CGGA) data accessed via GlioVis showed worsened prognosis in patients with high IGF2 expression (Supplementary Figure 1E).^18^ Collectively, these data suggested that oHSV therapy-induced intratumoral IGF2 expression and secretion activates IGF1R signaling, contributing to oHSV resistance.

### oHSV Induces IGF2 Secretion In Vitro and In Vivo Through Direct Binding of NFkB to IGF2 Promotor 3

**Figure 2.**
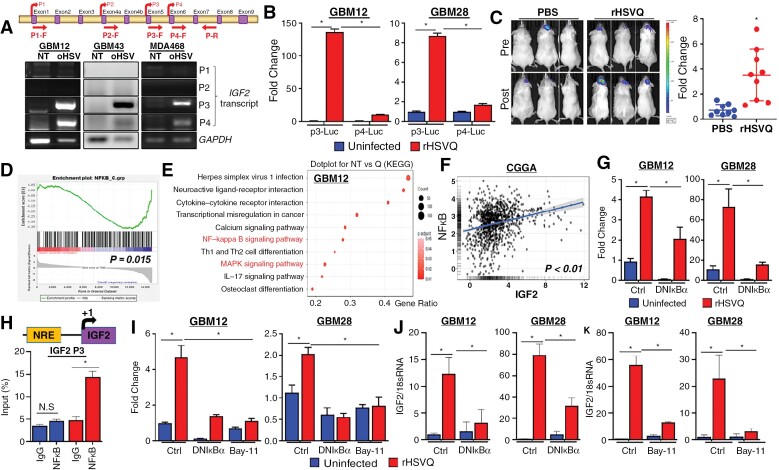
oHSV therapy induces IGF2 secretion in vitro and in vivo through direct binding of NFκB to IGF2 Promotor 3. (A) Schematic diagram of the four alternative IGF2 promoters, denoted P1-P4, and their exons and promoters. PCR primers for promoter-specific transcripts of IGF2 are described (top). GBM12, GBM43, and MDA468 cells were infected with or without rHSVQ (MOI = 0.1). Sixteen hours post viral infection, cells were harvested and the expression of IGF2 was tested by semi-quantitative RT-PCR. GAPDH expression was used for an internal control for gene expression. (B) Primary GBM12 cells stably expressing a firefly-luciferase reporter harboring either the IGF2 P3 (GBM12-IGF2P3-Luc) or the IGF2 P4 (GBM12-IGF2P4-Luc) reporter genes were infected with or without rHSVQ (MOI = 0.1) and luciferase activity was analyzed 24 hours later. The luciferase activity was normalized by protein concentration. Data shown are the mean ± S.D. of the relative change in IGF2 promoter-luciferase activity compared to unifected controls (*n* = 3/group). (C) The role of these promoters was examined in vivo by implanting GBM12-IGF2P3-Luc intracranially and then treating intratumorally with PBS or rHSVQ (5 × 10^5^ pfu). IGF2 promoter activation was measured by in vivo bioluminescence imaging eight hours before and after virus injection. Representative bioluminescence images of mice (left) and quantification of IGF2 promoter activity (right) revealed preferential IGF2P3 activation upon viral injection. Data shown represent the changes in luciferase activity pre- and post-rHSVQ injection (*n* = 9/group). (D-E) GSEA plot for NFκB signaling pathway (D) and KEGG pathway analysis showing the top 10 upregulated pathways (E) in the mRNA-Seq data (GBM12) prepresented in Figure 1. (F) There was a significant positive correlation between IGF2 and NFκB gene expression in glioma patients (*n* = 983) sampled in the CGGA. Log2-transformed mRNA expression data were obtained. IGF2 gene expression is plotted on the *x*-axis, while expression of NFκB genes is plotted on the *y*-axis. Linear regression estimates are expressed as a trend line. (G) rHSVQ infection induces NFκB activation. The primary GBM cells were transfected with a firefly-luciferase reporter harboring NFκB Response Elements (NREs; pGL3-NRE-fLuc), pGL3-TK-Renila luciferase (pGL3-TK-rLuc), and with either control pGL4.32 or dnIκBα-expressing plasmid (pGL4.32-dnIκBα). Twenty-four hours post-transfection, cells were infected with or without rHSVQ (MOI = 0.1) and luciferase activity was measured 24 hours later. The luciferase activity were normalized as a ratio of Firefly/Renilla Luciferase activity. Data shown are the mean ± S.D. of the relative change in NFκB-luciferase activity (*n* = 3/group). (H) Schematic diagram (top) depicts the promoter constructs of NREs within the IGF2P3 generated for ChIP analysis of promoter activity, which demonstrated binding of NFκB to the putative NRE within the IGF2P3 (bottom). Dominant-negative mutant of IκBα (dnIκBα) expression and direct NFκB inhibititor (Bay11-7082) treatment reversed rHSVQ treatment-induced NFκB activation and IGF2 gene expression, demonstrating that NFκB is both necessary and sufficient for IGF2 expression. GBM12 and GBM28 cells were co-transfected with either a control or IGF2P3-fLuc-expressing plasmid and pGL3-TK-rLuc plasmids. For the ectopic expression of a dnIκBα, 24 hours post-transfection, cells were transfected with control pGL4.32 or pGL34.2-dnIκBα plasmids. Twenty-four hours post-transfection, cells were infected with rHSVQ (MOI = 0.01 for GBM12 and MOI = 0.05 for GBM28) for 24 hours. For pharmacologic NFκB inhibition (Bay11-7082), cells were infected with rHSVQ (MOI = 0.01 for GBM12 and MOI = 0.05 for GBM28) 24-hour post-transfection and treated with 5 µM of Bay11-7082, 1-hour post viral infection, and cultured for 24 hours. (I) Luciferase activity was measured using a dual luciferase assay kit, normalized as a ratio of Firefly/Renila Luciferase activity. Data shown represent the fold change compared to uninfected controls (J-K) IGF2 expression was measured by qRT-PCR with molecular (dnIκBα overexpression) (J) and pharmacologic (Bay11-7082) inhibition (K) of NFκB as described above. IGF2 expression levels were normalized using 18S rRNA expression and presented as the fold change compared to unifected controls (*n* = 3/group). Data shown are the mean ± S.D. *P < .05, **P < .01, NS = not significant unless otherwise specified.

Transcription of the human IGF2 gene is regulated by four promoters (P1-P4), producing 4 distinct transcript variants in a spatially and temporally constrained manner (Figure 2A, top).^19–21^ To determine which promoter is responsible for the oHSV-triggered induction of IGF2 gene expression, semi-quantitative RT-PCR was performed with primer sets corresponding to each of those 4 transcript variants. Among the 4 variants, a transcript variant governed by P3 was the most abundant species in the rHSVQ-infected cells, followed by a transcript produced by P4, while no apparent P1 or P2 transcripts were observed (Figure 2A, bottom). To confirm the promoter activity at P3 and P4 upon rHSVQ infection, GBM cells stably expressing the firefly-luciferase reporter gene under the control of either IGF2 P3 (IGF2P3-Luc) or IGF2 P4 (IGF2P4-Luc) were infected with or without rHSVQ (MOI = 0.1) for 24 hours. Consistent with the semi-quantitative RT-PCR results, rHSVQ infection significantly increased the luciferase activity in both IGF2P3-Luc- and IGF2P4-Luc-expressing GBM cells, but was markedly higher in IGF2P3-Luc cells compared to IGF2P4-Luc cells (Figure 2B). For in vivo validation, mice bearing intracranial GBM12-IGF2P3-Luc or GBM12-IGF2P4-Luc tumors were intratumorally treated with rHSVQ or PBS and monitored by IVIS imaging, as previously described (Figure 2C).^1,22,23^ A 4.8-fold significant increase in viral luminescence intensity was observed in mice implanted with GBM12-IGF2P3-Luc (Figure 2C), while there was no difference observed in the mice implanted with GBM12-IGF2P4-Luc (Supplementary Figure 2A), suggesting that the IGF2 induction by oHSV therapy occurs primarily at IGF2 P3.

To further characterize the mechanism by which IGF2 transcription is induced, we performed gene set enrichment analysis of our mRNA-Seq data which showed a significant enrichment of NFκB (Figure 2D), signal transducer and activator of transcription 3 (STAT3), and GATA-binding factor 2 (GATA2) (Supplementary Figure 2B), all of which have been shown to enhance transcriptional activation of IGF2.^21,24–28^ Kyoto Encyclopedia of Genes and Genomes enrichment analysis of the mRNA-Seq data also identified NFκB signaling pathway as one of the most upregulated following rHSVQ treatment (Figure 2E and Supplementary Figure 2C). Additionally, there was a significant positive correlation between expression of IGF2 and NFκB (Figure 2F), GATA2 and STAT3 (Supplementary Figure 2D) in brain tumor patients sampled in the CGGA dataset. However, GATA2 gene expression was not increased in rHSVQ-infected GBM cells and transient GATA2 knockdown did not prevent up-regulation of IGF2 gene expression (Supplementary Figure 2E). Similarly, promoter activity of STAT3 was not increased in rHSVQ-infected GBM cells (Supplementary Figure 2F). In contrast, an NFκB reporter assay showed a significant increase in promoter activity after rHSVQ infection, which was abolished by molecular inhibition of NFκB through ectopic expression of a dominant-negative mutant of IκBα (dnIκBα) (Figure 2G). These data suggest that NFκB is responsible for the transcriptional activation of IGF2 following rHSVQ treatment.

Based on TRANSFAC analysis (http://genexplain.com/transfac/), a putative NFκB response element site was identified in the IGF2 P3 region (Figure 2H, top). Chromatin immunoprecipitation (ChIP) assay using an anti-NFκB antibody in GBM12 cells revealed a significant recruitment of NFκB to the IGF2 P3 after rHSVQ treatment (Figure 2H). Additionally, the rHSVQ-dependent activation of IGF2 P3 (Figure 2I) and IGF2 gene expression (Figure 2J and 2K) were abolished by both molecular inhibition of NFκB through ectopic expression of dnIκBα (Figure 2J) or pharmacologic NFκB inhibitor (Bay11-7082) (Figure 2K). Collectively, these results suggested that oHSV infection up-regulates the expression of IGF2 through transcriptional activation by NFκB via its direct binding to the NFκB response element in the IGF2P3.

### Inhibition of IGF2 Enhances Therapeutic Efficacy of oHSV In Vitro and In Vivo

**Figure 3.**
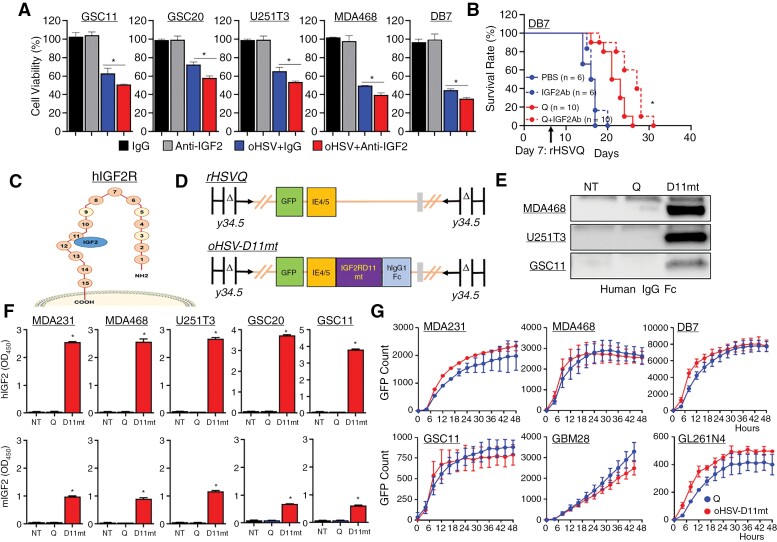
The novel next-generation oHSV, oHSV-D11mt, secretes an IGF2R domain 11 decoy receptor with specificity for IGF2 without altering viral kinetics. (A) Various cancer cells were infected with or without rHSVQ at an MOI of 0.01 or 0.05, and then treated with 20 µg/mL of either IgG isotype control or an anti-IGF2 antibody 1 hour later. Seventy-two hours post-infection, cell viability was measured by a standard MTT assay. Data represent the mean % cell viability relative to uninfected cells ± S.D. (*n* = 3/group). *, *P* < 0.05.(B) Kaplan–Meier survival curve of mice bearing intracranial DB7 murine BCBM tumors and treated intratumorally with PBS or rHSVQ (Q) (5 × 10^5^ pfu) 9 days post tumor implantation and then treated with 20 µg/mouse of IgG isotype control or an anti-IGF2 antibody 3 times a week. Animal numbers used are described inside of survival curve. (C) Illustration of IGF2R depicting binding specificity of domain 11 for IGF2. (D) The genomic structure of F-strain HSV-1 shows doubly deleted **γ**34.5 genes, a disrupted ICP6 gene, and an inserted eGFP transgene within the control rHSVQ (top, Q) and oHSV-D11mt (bottom, D11mt). Our next-generation oHSV, oHSV-D11mt, contains both an eGFP and an IGF2RD11mt-hIgGFc fusion transgene. (E) Using culture media (CM) collected from MDA468, U251T3, and GSC11 cells infected with either rHSVQ- and oHSV-D11mt for 16 hours, secreted IGF2RD11mt was probed by western blot analysis using human IgGFc antibody. (F) Using the CM from rHSVQ- and oHSV-D11mt-infected various BC and GBM cells for 16 hours, specific binding affinity of IGF2RD11mt to human IGF2 (top) and murine IGF2 (bottom) was quantified by ELISA using a secondary HRP-conjugated anti-human IgGFc antibody. (H) Comparison of viral spread/kinatics in cultures of the indicated BC and GBM cells infected with rHSVQ and oHSV-D11mt, showed no difference in viral replication. The indicated BC and GBM cells were infected with rHSVQ or oHSV-D11mt and viral GFP expression was monitored every 2 hours for 48 hours utilizing the Cytation 5 live imaging system. Viral GFP count was quantified and graphed as an average of 3 wells per treatment group. Data shown are average counts of GFP positive cells ± SD over time. *P < .05.

Next, we evaluated the therapeutic potential of IGF2 blockade in conjunction with oHSV therapy. In vitro, rHSVQ-induced cytotoxicity was enhanced when combined with an IGF2 neutralizing antibody in all tested GBM and BC cells compared to either therapeutic or monotherapy (Figure 3A). Further, intratumoral injection of the anti-IGF2 antibody with rHSVQ significantly enhanced survival of DB7 BCBM tumor-bearing mice (median survival of 27 days) compared to anti-IGF2 antibody (median survival of 16.5 days, *P* < .001) or rHSVQ monotherapy (median survival of 22 days, *P  = .0041;*Figure 3B). Importantly, systemic delivery of an IGF2-neutralizing antibody failed to improve therapeutic efficacy of rHSVQ in intracranial 005 murine glioma and DB7 BCBM tumors, likely due to the inability of the antibody to penetrate the blood-brain barrier (BBB; Supplementary Figure S3A). Therefore, local delivery of IGF2-specific blockade is crucial for improving the therapeutic efficacy of oHSVs in intracranial tumors.

### The Novel Next-Generation oHSV, oHSV-D11mt, Secretes an IGF2R Domain 11 Decoy Receptor With Specificity for IGF2 Without Altering Viral Kinetics

Given the poor distribution of systemically administered IGF2-specific inhibitors, we aimed to design a next-generation oHSV capable of local inhibition of IGF2 to abrogate the resistance conferred by IGF2 secretion. Because the IGF2R lacks tyrosine kinase activity, it acts predominantly as an antagonist for circulating IGF2.^29^ Among the 15 extracellular domains on IGF2R, IGF2 binds to domain 11 (Figure 3C), which we hypothesized it could be exploited as a decoy receptor for IGF2. In addition, a previously described mutation in domain 11 increases the binding affinity to IGF2 without altering its specificity.^30^ Thus, we generated a novel next-generation oHSV, hereafter referred to as “oHSV-D11mt,” designed to express and secrete the mutated domain 11 of IGF2R fused to the human IgG Fc domain (IGF2RD11mt) capable of acting as a decoy receptor within the TME. We accomplished this by inserting the sequence within the HSV-1 F-strain backbone using HSVQuick-technology (Figure 3D).^15^ To confirm the secretion of IGF2RD11mt, MDA468, U251T3, and GSC11 cells were infected with rHSVQ or oHSV-D11mt, and culture media was collected 14 hours later and subjected to western blot (Figure 3E). The binding affinity of the secreted IGF2RD11mt was further determined by sandwich ELISA using plates pre-coated with purified human (hIGF2) or murine IGF2 (mIGF2) and an anti-hIgGFc detection antibody. The secreted IGF2RD11mt demonstrated high affinity for IGF2 and no detectable binding to hIGF1, indicating specificity of IGF2RD11mt to hIGF2 (Figure 3F, top and Supplementary Figure S3B). Notably, IGF2RD11mt also bound to mIGF2, although the efficiency was approximately 3 times lower (Figure 3F, bottom), indicating that IGF2RD11mt also can serve as a decoy receptor for mIGF2 secreted from host TME cells following rHSVQ treatment in a syngenic mouse xenograft model. Further, a real-time live cell imaging revealed no significant changes in viral kinetics/replication between control rHSVQ- and oHSV-D11mt-infected cells (Figure 3G). Additionally, the neutralization of IGF2 did not affect viral replication or propagation in all cancer cells tested in vitro (Supplementary Figure S3C-D). Overall, these data confirm that the secreted IGF2RD11mt serves as a decoy receptor against both human and murine IGF2 without altering the efficiency of viral infection and replication.

### oHSV-D11mt Enhances Direct Tumor Cell Killing and Immune Cell-Mediated Cytotoxicity In Vitro

**Figure 4.**
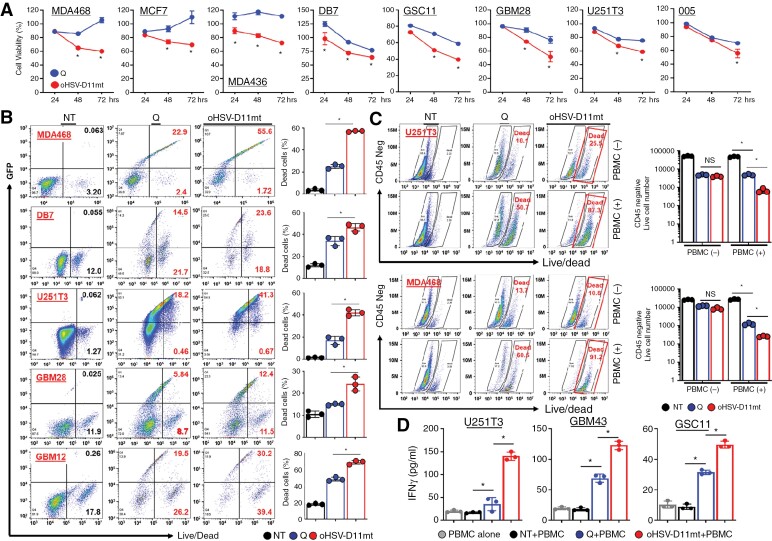
oHSV-D11mt enhances direct tumor cell killing and immune cell-mediated cytotoxicity in vitro. (A) Various BC and GBM cells were infected with control rHSVQ or oHSV-IGF2RDmt (MOI = 0.05 ~ 0.1) and cell viability was measured by MTT assay 24, 48, and 72 hours post-viral infection. Data represent the mean % cell viability relative to uninfected cells ± SD for each group (*n* = 3/group). (B) Human BC and GBM cells infected with rHSVQ or oHSV-D11mt (MOI = 0.05 ~ 0.1) for 48 hours were stained with live/dead fixable aqua cell stain and then analyzed by flow cytometry. The results are illustrated as a representative scatter plot (left) and the percentage of dead cells (right). (C-D) U251T3 or MDA468 cells were infected with rHSVQ or oHSV-D11mt and overlaid with PBMCs. Five days after co-culture, CM and cells were collected and cells were stained with a CD45 antibody and live/dead fixable aqua cell stain and then analyzed by flow cytometry (C). Data shown are a representative scatter plot. (D) Using CM collected from (C), human IFNγ secretion was quantified by ELISA. *, *P* < .05.

Cytotoxicity of oHSV-D11mt was assessed in vitro by infecting glioma or BC cells with either rHSVQ or oHSV-D11mt at various MOIs for 24, 48, and 72 hours and then subjected to a standard MTT cell viability assay. Although viral kinetics/propagation appeared similar (Figure 3G), oHSV-D11mt significantly enhanced tumor cell killing compared to rHSVQ (Figure 4A). This greatly enhanced cytotoxicity was further verified by live/dead staining, as previously described.^1,31^ In all tested cells, oHSV-D11mt increased the population of dead cells compared to rHSVQ (Figure 4B).

Several in vitro and in vivo studies have shown that inhibition of IGF1R signaling induces an antitumor immune response in BC and GBM models.^9–12^ To assess whether oHSV-D11mt can activate immune cells and induce cytotoxicity towards infected cancer cells, we performed an in vitro co-culture assay of rHSVQ- or oHSV-D11mt-infected glioma and BC cells with peripheral blood mononuclear cells (PBMCs). GBM or BC cells were infected at 0.01 MOI and co-cultured with PBMCs at 1:5 ratio of tumor cells to PBMCs for five days. Interestingly, live/dead staining and quantification of IFNγ revealed that oHSV-D11mt infection markedly enhanced cytotoxicity and IFNγ secretion when co-cultured with PBMCs (Figure 4C-D). Collectively, these data suggest that oHSV-D11mt infection increases direct virus-mediated tumor cell killing as well as immune cell-mediated tumor cell killing.

### oHSV-D11mt Enhances Therapeutic Efficacy in Orthotropic Mouse Models of GBM and BCBM by Evoking Lasting Antitumor T-cell Immunity

**Figure 5.**
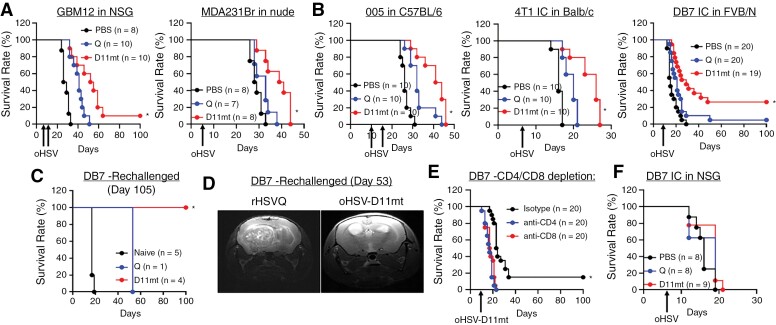
oHSV-D11mt treatment improves mice survival in GBM- and BCBM-bearing immunocompromised and immunocompetent mice. (A–B) Kaplan–Meier survival curve of orthotropic intracranial mouse models of human patient-derived primary GBM12 and MDA231Br human BC cells in immunocompromised mice (A) and 005 murine glioma, 4T1 and DB7 murine BC cells in immunocompetent mice (B) treated intra-tumorally with PBS, rHSVQ, or oHSV-D11mt (5 × 105 pfu). (C) Long-term survivors of DB7 BCBM tumors treated with rHSVQ (*n* = 1) and oHSV-D11mt (*n* = 4) in part A were re-challenged with a secondary tumor implantation in the opposing hemisphere without re-treatment. While all control age-matched naive mice died 19 days post-implantation, the rHSVQ-treated long-term survivor died 53 days post-tumor implantation and all oHSV-D11mt-treated long-term survivors fully rejected the tumors as seen in MRI (D). (E) Intracranial DB7 murine BCBM tumor-bearing FVB/N mice were treated with or without rHSVQ as described above. We then depleted either CD4 + or CD8 + T cells by intraperitoneal (IP) administration of depleting antibodies (IgG isotype control, anti-CD4, or anti-CD8) 2, 4, 7, and 10 days post viral injection (*n* = 20/each group). *P < .05 compared with CD4 or CD8 depletion. (F) Kaplan–Meier survival curves of immunodeficient NSG mice implanted with intracranial DB7 murine BCBM tumors and treated intra-tumorally with PBS, rHSVQ, or oHSV-D11mt (5 × 10^5^ pfu) 7 days later, revealing a reversal of the survival benefit observed in syngenic immunocompetent models. Data are presented as means ± SD with *P < .05.

Next, we evaluated the antitumor efficacy of oHSV-D11mt in both immunocompromised (e.g. GBM12 tumor-bearing NSG mice and MDA231Br BCBM tumor-bearing athymic nu/nu) and immunocompetent (e.g. 005 glioma in C57BL6, 4T1 BCBM in BALB/c, and DB7 BCBM in FVB/N) intracranial mouse xenograft models. Kaplan–Meier survival curves of the GBM12 tumor-bearing mice showed a significantly improved survival after oHSV-D11mt treatment (median 52 days) compared to the mice treated with rHSVQ or PBS (median 40 or 26 days, respectively, *P* < .0001; Figure 5A). Similarly, the median survival of mice bearing MDA231Br tumors was improved to 40 days with oHSV-D11mt treatment, compared to 33 days in mice treated with rHSVQ (*P* = .0105; Figure 5A). The median survival in syngeneic murine GBM and BCBM tumor-bearing mice was also significantly improved after treatment with oHSV-D11mt compared to rHSVQ (Figure 5B). Interestingly, approximately 26.3% of oHSV-D11mt-treated DB7 BCBM tumor-bearing mice survived over 100 days, while the control mice treated with rHSVQ showed no significant improvement in survival compared to PBS-treated mice (Figure 5B).

To assess the effect of oHSV-D11mt on long-term antitumor immune memory, the surviving mice were re-challenged with a second tumor implantation in the opposite brain hemisphere. Age-matched naïve mice had a median survival of 19 days, while the one surviving mouse previously treated with rHSVQ had a median survival of 53 days following reimplantation (Figure 5C). Surprisingly, all re-challenged oHSV-D11mt-treated surviving mice demonstrated complete rejection of subsequent tumor growth without re-treatment (Figure 5C). The complete tumor rejection was further confirmed by magnetic resonance imaging at 53 days post-tumor reimplantation (Figure 5D). These data strongly suggest that oHSV-D11mt treatment successfully developed an adaptive antitumor memory response in 26.3% of the mice treated.

To conclusively demonstrate that enhanced T cell recruitment by oHSV-D11mt is critical to its enhanced efficacy, mice were separated into three cohorts for T cell depletion receiving either anti-CD4, anti-CD8, or isotype control antibodies (i.e. IgG) post-virus injection. When DB7 tumor-bearing mice were treated with oHSV-D11mt without T cell depletion, approximately 15% (3 out of 20) mice achieved long-term survival (Figure 5E). In contrast, the mice treated with oHSV-D11mt and T cell depleting antibodies showed no survival benefit (Figure 5E), indicating the critical role of T cell immunity in antitumor efficacy of oHSV-D11mt. Similarly, immunocompromised DB7 BCBM tumor-bearing NSG mice exhibited no survival benefit from oHSV-D11mt treatment (Figure 5F), suggesting that the augmented T-cell immunity underlies the enhanced efficacy of oHSV-D11mt in vivo.

### oHSV-D11mt Significantly Increases CD8 + Tumor-Infiltrating T Lymphocyte Recruitment Without Increased Infiltration of Neutrophils/PMN-MDSCs

**Figure 6.**
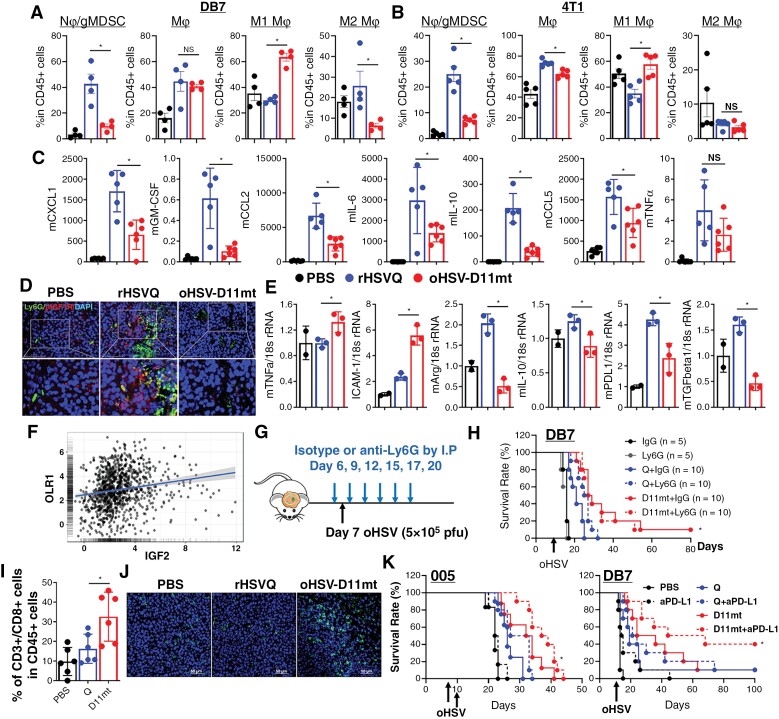
oHSV-D11mt significantly increases CD8 + Tumor-Infiltrating T lymphocyte recruitment without increased infiltration of neutrophils/PMN-MDSCs in orthotropic models of BCBM. (A–B) Intracranial DB7 (A) and 4T1 (B) BCBM tumor-bearing mice were injected with PBS, rHSVQ, or oHSV-D11mt (5 × 10^5^ pfu) 10 days post-tumor implantation. Tumor-bearing brain hemispheres were collected two days post-virus injection and analyzed for CD11b^high^/CD45+/Ly6G + gMDSC and CD11b^high^/CD45 + monocyte-derived macrophage infiltration and activation by flow cytometry. (C) Bioplex Luminex assay using tumor lysates collected from DB7 BCBM tumor-bearing mice treated with PBS, rHSVQ, or oHSV-D11mt. Cytokine measurements (pg/mL) were normalized via Box-Cox transformations and Pearson’s correlation coefficient was calculated for each pair of the cytokines to generate a correlation matrix. (D) Immunofluorescence Staining of Ly6G + neutrophils/PMN-MDSC infiltration and activation of IGF1R in DB7 BCBM tumor-bearing mice treated as above revealed a marked reduction in infiltration of neutrophils/PMN-MDSCs (Ly6G+, green) and a concurrent activation/phosphorylation of IGF1R (p-IGF1R, red). (E) Ly6G + neutrophil/PMN-MDSCs were isolated from tumor-bearing hemispheres two days after viral treatment of DB7 BCBM tumors treated with PBS, rHSVQ, or oHSV-D11mt (5 × 10^5^ pfu) ten days after implantation. Gene expression of markers associated with pro-inflammatory (N1) neutrophils (TNFα and ICAM-1) and anti-inflammatory (N2) neutrophils (Arg1, IL-10, PD-L1, and TGFβ) were measured by qRT-PCR. All gene expression levels were normalized using 18S expression and presented as the fold change compared to PBS controls. *P < .05 compared to rHSVQ. (F) There was a significant correlation between IGF2 and OLR1 gene expression in glioma patients (*n* = 983) sampled in the CGGA. Log2-transformed mRNA expression data were obtained. IGF2 gene expression is shown as *x*-axis, while expression of OLR1 genes is shown on *y*-axis. Linear regression estimates are shown as a trend line. (G-H) (G) Schematic diagram of treatment schedule. (H) Intracranial DB7 murine BCBM tumor-bearing FVB/N mice were treated intratumorally with PBS, rHSVQ, or oHSV-D11mt then with an isotype control IgG or anti-Ly6G gMDSC depleting antibody by IP injection as described in the experimental scheme (G). Data shown are Kaplan–Meier survival curves of animals in each group (PBS + Isotype, *n* = 5; PBS + anti-Ly6G, *n* = 5; rHSVQ + anti-Ly6G, *n* = 10; oHSV-D11mt + anti-Ly6G, *n* = 10). (I) Flow-cytometry analysis of CD4 + and CD8 + TILs collected from DB7 BCBM tumor-bearing hemispheres receiving PBS, rHSVQ, and oHSV-D11mt treatment revealed no change in CD4 + helper T cells and a significant increase in CD8 + cytotoxic T lymphocytes (CTLs, n = 6/group). Data shown are the mean ± SEM. (J) Brain tumor tissue from (D) were stained with a CD8 (green) antibody, revealing a marked increase in CD8 + CTLs in oHSV-D11mt-treated mice. (K) oHSV-D11mt synergized with adjuvant T cell activation through immune checkpoint blockade. We administered an adjuvant anti-PDL1 antibody as described in the experimental scheme in (G) in the 005 glioma and DB7 BCBM model (*n* = 10/group). All data are presented as means ± SEM with *P < .05.

Next, we further explored the mechanisms underlying the antitumor immunity induced by oHSV-D11mt in vivo. First, analytical multi-color flow cytometry was performed on immune cells harvested from DB7 BCBM tumor-bearing brain hemispheres at 2 days post-virus injection. As expected, all the mice treated with either rHSVQ or oHSV-D11mt had increased tumor-infiltrating macrophages and Ly6G + neutrophils/PMN-MDSCs when compared to the mice treated with PBS (Figure 6A). However, oHSV-D11mt-treated mice showed significantly decreased recruitment of Ly6G + neutrophils/gMDSCs compared to rHSVQ treatment. In addition, M1 macrophages were enriched in mice treated with oHSV-D11mt compared to rHSVQ (Figure 6A). A similar pattern was observed when the same experiment was carried out in intracranial 4T1 BCBM tumor-bearing mice (Figure 6B). Analysis of DB7 BCBM tumor lysates using a murine bio-plex cytokine panel showed that rHSVQ treatment induced secretion of chemokines attracting Ly6G + neutrophils/PMN-MDSCs, such as CXCL1, GM-CSF, IL-6, IL-10 and MCP1 (Figure 6C and Supplementary Table S4), which was significantly reduced by oHSV-D11mt treatment. Additionally, immunofluorescence staining of DB7 BCBM tumors revealed that oHSV-D11mt treatment effectively abrogated the increased recruitment of immunosuppressive Ly6G + neutrophils/PMN-MDSCs and IGF1R activation in vivo (Figure 6D). Similarly, local anti-IGF2 antibody treatment significantly decreased oHSV therapy-induced recruitment of Ly6G + neutrophils/PMN-MDSCs in DB7 BCBM tumor-bearing mice (Supplementary Fig. S4). Moreover, qRT-PCR analysis of Ly6G + myeloid cells purified from DB7 BCBM tumors treated with PBS, rHSVQ, or oHSV-D11mt showed that oHSV-D11mt significantly increased the number of neutrophils expressing pro-inflammatory cytokines (TNFα and ICAM1) and decreased Ly6G + neutrophils/PMN-MDSCs expressing anti-inflammatory cytokines (Arg1, IL-10, PD-L1, and TGFβ; Figure 6E). Analysis of CGGA patient data revealed a positive correlation between IGF2 and OLR1 (LOX-1), a PMN-MDSC-specific marker, providing a degree of external validity to these findings (Figure 6F).^32^ Furthermore, depletion of Ly6G + neutrophils/PMN-MDSCs markedly enhanced the therapeutic efficacy of rHSVQ, but did not alter the therapeutic efficacy of oHSV-D11mt (Figure 6G-H). Collectively, these findings strongly suggest that oHSV therapy-induced IGF2 secretion recruits circulating Ly6G + neutrophils/PMN-MDSCs and generates an immunosuppressive TME, compromising viro-immunotherapy. oHSV-D11mt alleviates these resistant mechanisms, promoting an inflammatory TME capable of producing lasting antitumor immunity.

Of critical importance, a significantly higher level of CD3^+^/CD8^+^ tumor-infiltrating T lymphocytes (TILs) was observed in the mice treated with oHSV-D11mt compared to the mice treated with rHSVQ, suggesting that enhanced activation of adaptive T-cell immunity plays a key role in the observed enhanced therapeutic efficacy (Figure 6I–J). However, despite the greatly enhanced survival observed in oHSV-D11mt treatment in preclinical models (Figure 5B), only 35% of oHSV-D11mt-treated tumors were completely eradicated. We, therefore, hypothesized that the CD3^+^/CD8^+^ TILs induced by oHSV-D11mt require further activation by ICB (ie, PD-L1) to achieve maximum antitumor efficacy. Kaplan–Meier survival curves revealed that a significant survival benefit was achieved in the mice treated with oHSV-D11mt and anti-PD-L1 compared to the mice treated with either oHSV-D11mt or anti-PD-L1 alone in both 005 murine glioma and DB7 BCBM tumor-bearing immunocompetent mice (Figure 6K). Taken together, these data suggest that one mechanism of enhanced therapeutic efficacy of oHSV-D11mt involves increased recruitment of cytotoxic TILs, which can be further synergized with ICB.

## Discussion

Numerous oHSV treatments have progressed to clinicals, demonstrating their safety and therapeutic advantages. However, only a small fraction of patients treated with oHSVs experience meaningful clinical responses, and their therapeutic efficacy is often limited related to the tumor cells and cells within the TME. Elucidating mechanisms by which oHSV-treated tumors develop resistance to oHSVs is therefore critical in maximizing therapeutic efficacy. In this study, we provide robust evidence that oHSV therapy induces the secretion of IGF2, supporting tumor regrowth and maintenance of an immunosuppressive TME following viral clearance.

Multiple Receptor tyrosine Kinases, including EGFR and PDGFR, have been validated as therapeutic targets for GBM and BCBM, yet these RTK inhibitors have had limited success in the clinic.^1,5,33–38^ Additionally, increased IGF1R expression/activation is known to mediate resistance to RTK inhibitors, chemotherapy, and radiotherapy. Therefore, therapeutic strategies combining IGF1R inhibition with various therapeutic modalities are currently under investigation, with promising preclinical results. In addition, in 2020, TEPEZZA® (teprotumumab-trbw), a fully human monoclonal antibody and a targeted inhibitor of the IGF1R, was FDA-approved for the treatment of Thyroid Eye Disease.^39^ While others have proposed the use of oHSVs in combination with other RTK-targeting therapies (EGFR and VEGFR inhibitors), to our knowledge this is the first approach to evaluate the preclinical impact of IGF1R pathway inhibition in conjunction with oHSV therapy. Our data demonstrate that oHSV therapy induces activation of the IGF1R signaling pathway, potentially sensitizing virus-infected tumor cells to IGF1R-targeting drugs. Further, the oHSV-D11mt sensitized tumors to the treatment of ICB (anti-PD-L1 therapy) and improved the antitumor efficacy of either oHSV-D11mt or anti-PD-L1 monotherapy, extending the overall survival in GBM and BCBM tumor-bearing mice. Thus, our results provide promising preclinical evidence supporting the future translation of combination therapy with oHSV therapy and IGF1R inhibitors and/or ICB in a clinical setting.

Efficient delivery of IGF2 blockade to the brain is critical to the therapeutic efficacy of oHSV for primary and metastatic brain tumors (Figure 3B and Supplementary Figure S3A). Single-cell RNA-seq (scRNA-Seq) data analyzing GBM patient samples shows that IGF2 is mostly expressed in neoplastic and vascular cells, not in neuronal cells in which oHSVs do not replicate.^40^ Additionally, IGF2 expression is significantly increased directly around the oHSV replication area (Figure 1H). oHSV-D11mt treatment secretes IGF2RD11mt locally during active replication, efficiently mitigating the tumor-supportive effects of oHSV-induced IGF2 secretion and greatly enhancing therapeutic efficacy. Our engineered oHSV-D11mt represents an elegant solution to this mechanism of oHSV resistance, eliminating the need for additional IGF2-IGF1R inhibitors that are unable to adequately traverse the blood-brain barrier (BBB). It is our belief that oHSV-D11mt would reduce the cost burden associated with treating GBM and metastatic brain tumors, which often require multimodal therapy, ultimately improving patient outcomes and access to care.

A plethora of studies have established that the genomic landscape and immune cell distribution in brain metastases derived from distant extracranial primary tumors, such as BC and melanoma, differ dramatically from primary brain tumors.^40–42^ For example, gliomas contain an abundance of TAMs and fewer T cells, while BCBM tumors have fewer TAMs, greater numbers of lymphocytes, and a marked accumulation of neutrophils, shown to play tumor-supportive and immunosuppressive roles within the TME.^41^ The difference in tumor cell biology and TME characteristics between glioma and BCBM likely underlies the observed differences in treatment response. Interestingly, while treatment of both BCBM and GBM tumor-bearing mice with rHSVQ significantly increased neutrophil/PMN-MDSC infiltration into the TME, a significantly higher number of neutrophils/PMN-MDSCs were found in the BCBM tumor-bearing mice (Figure 6A-B) compared to glioma-bearing mice (Supplementary Figure S5A and Supplementary Table S5). Depletion of neutrophils/PMN-MDSCs using an anti-Ly6G antibody markedly enhanced the therapeutic efficacy of rHSVQ in DB7 murine BCBM tumor-bearing mice (Figure 6H), while no such effect was observed in 005 murine glioma tumor-bearing mice (Supplementary Figure S5B). Moreover, oHSV-D11mt treatment significantly increased the number of anti-tumoral/pro-inflammatory neutrophils and simultaneously decreased pro-tumoral/anti-inflammatory neutrophils (Figure 6E). These findings strongly suggest that oHSV-D11mt effectively reduces feedback immunosuppression after oHSV therapy by inhibiting infiltration and phenotypically regulating neutrophils/PMN-MDSCs. Additionally, while a single dose of oHSV-D11mt significantly improved survival in BCBM tumor-bearing mice, a second dose was required for glioma-bearing mice, indicating that the BCBM tumors are more susceptible to oHSV-D11mt than GBM (Figure 5A-B and Supplementary Figure S6A). Therefore, it would be intriguing to investigate mechanisms by which oHSV-induced IGF2 modulates the tumor and TME differently in GBM and BCBM tumors. In addition, our group is currently investigating the functional role of neutrophils/PMN-MDSCs in the context of oHSV therapy and the impact of oHSV-induced IGF2 secretion in regulating neutrophils/PMN-MDSCs within the TME.

IGF1 is a ubiquitously expressed growth factor which functions as a key regulator of neurogenesis and synaptogenesis,^43^ and is thought to be essential in numerous neural functions. IGF2, conversely, has minimal physiologic relevance in neural function, is highly expressed in cancer cells, and is associated with poor clinical outcomes.^44^ Cancer stem cells (CSCs) are thought to be responsible for tumor recurrence and drug resistance.^45,46^ Accumulating evidence suggests that IGF2 is vital in promoting and maintaining CSCs, which are thought to be responsible for tumor recurrence and drug resistance.^47,48^ Indeed, IGF1R signaling is highly upregulated in CSC-enriched populations and IGF2-PI3K signaling induces gene expression of stemness transcription factors and IGF2 itself.^47,48^ Although our data clearly shows that oHSV-D11mt inhibits neutrophil/PMN-MDSC infiltration and activation T cells, oHSV-D11mt may have an even greater effect on CSCs due to their reliance on IGF1R signaling to maintain their stem-like character. Satoro et al. recently demonstrated up-regulation of the IGF1R signaling in glioma stem cells following repeated radiation therapy (RTx), inducing adaptive radioprotection and escape from RTx-induced cytotoxicity.^47,48^ Further, the combination treatment of radio-resistant glioma stem cells with RTx and IGF1R inhibition resulted in a robust increase in radiosensitivity, suggesting that the specific inhibition of IGF1R signaling is a promising strategy to reverse RTx resistance and improve patient survival. Therefore, future work elucidating the efficacy and impact of oHSV-D11mt in combination with RTx will lend further insight into the therapeutic management of intracranial neoplasms which ubiquitously receive RTx.

While depletion of Ly6G + myeloid cells partially reversed the therapeutic efficacy of oHSV-D11mt in human MDA231Br BCBM tumor-bearing immunocompromised mice (Supplementary Figure S6B), it completely abrogated therapeutic efficacy in syngenic immunocompetent BCBM tumor models (Figure 6H). We argue, therefore, that the enhanced therapeutic efficacy of oHSV-D11mt in BCBM is achieved both by reducing tumor cell growth and Ly6G + neutrophil/PMN-MDSCs infiltration in vivo. Notably, oHSV-D11mt binds mouse IGF2 more weakly than human IGF2 (Figure 3G), resulting in low cytotoxicity against mouse cancer cells (Figure 4A). Moreover, human-specific rHSVQ replicates poorly in mice, resulting in reduced IGF2RD11mt secretion in mouse cells compared to human cells. Accumulating data have demonstrated that the duration of oHSV persistence within the tumor and TME is significantly correlated with immune cell recruitment.^1^ Therefore, since we tested oHSV therapy-induced immunosuppression/activation in murine brain and BCBM tumor models, our results do not exclude the possibility that the oHSV therapy-induced immunosuppression could be related to limited viral replication and spread in murine tumor models. Future studies in our group will evaluate the efficacy of oHSV-D11mt in humanized mouse models to more accurately simulate the ability of our novel therapeutic to recruit human adaptive immunity.

In conclusion, in this work, we identified and elucidated a novel mechanism of resistance to oHSV therapy by which oHSV therapy induces cancer–TME cell communication via NFκB-dependent IGF2 expression and secretion. IGF2 acts to enhance IGF1R signaling within both the tumor and TME cells, supporting tumor regrowth and inducing feedback immunosuppression. Targeting IGF2-IGF1R signaling, therefore, represents a promising strategy to overcome resistance to oHSV therapy. Furthermore, we designed and validated a novel oHSV-D11mt capable of secreting a modified domain 11 of the IGF2R that acts to neutralize IGF2 within the TME and provide strong evidence that local IGF2 inhibition in conjunction with viro-immunotherapy enhances therapeutic efficacy. Finally, we assert that innovative approaches to modulating the TME, such as those described here, will be crucial in overcoming the limited clinical success of viro-immunotherapy in the treatment of intracranial neoplasms, ultimately advancing the field of neuro-oncology as a whole.

## Supplementary material

Supplementary material is available online at *Neuro-Oncology* (https://academic.oup.com/neuro-oncology).

noae105_suppl_Supplementary_Materials

noae105_suppl_Supplementary_Tables_S1-S3_S5

noae105_suppl_Supplementary_Data

## Data Availability

All data are available in the main text or the supplementary materials.
