# Supplementary material for: Targeting IGF2 to reprogram the tumor microenvironment for enhanced viro-immunotherapy
Source: Neuro Oncol. 2024 Jun 10;26(9):1602–16. doi: 10.1093/neuonc/noae105 (PMC11376453; doi:10.1093/neuonc/noae105)
Supplement: noae105_suppl_Supplementary_Materials [file noae105_suppl_supplementary_materials.docx]

**Targeting IGF2-IGF1R Signaling to Reprogram the Tumor Microenvironment for Enhanced Viro-Immunotherapy**

Min Hye Noh^1*^, Jin Muk Kang^1,9*^, Alexandra A. Miller^1,2^, Grace Nguyen^1^, Minxin Huang^1^, Ji Seon Shim^1^, Alberto J Bueso-Perez^1^, Sara Murphy^1,3^, Kimberly A. Rivera-Caraballo^1,3^, Yoshihiro Otani^1,4^, Eunju Kim^5^, Seung-Hee Yoo^5^, Yuanqing Yan^1,6^, Yeshavanth Banasavadi-Siddegowda^7^, Hiroshi Nakashima^8^, E. Antonio Chiocca^8^, Balveen Kaur^3^, Zhongming Zhao^9^, Tae Jin Lee^1,2#^, and Ji Young Yoo^1,2#^

^1^Department of Neurosurgery, McGovern Medical School, The University of Texas Health Science Center at Houston, Houston, TX, 77030, USA. ^2^MD Anderson Cancer Center UTHealth Graduate School of Biomedical Science, Houston, TX 77225, USA. ^3^Georgia Cancer Center and Department of Pathology, Medical College of Georgia, Augusta University, 1410 Laney Walker Blvd, CN-3311, Augusta, GA 30912, USA. ^4^Department of Neurological Surgery, Okayama University Graduate School of Medicine, Dentistry, and Pharmaceutical Sciences, 2-5-1 Shikata-cho, Kita-ku, Okayama, 700-8558, Japan. ^5^Department of Biochemistry, McGovern Medical School, The University of Texas Health Science Center at Houston, Houston, TX, 77030, USA. ^6^Department of Surgery, Northwestern University Feinberg School of Medicine, Chicago, IL, 60611, USA. ^7^Surgical Neurology Branch, National Institute of Neurological Disorders and Stroke, National Institutes of Health, Bethesda, MD, 20852, USA. ^8^Department of Neurosurgery, Brigham and Women's Hospital and Harvard Medical School, Boston, MA, 02115, USA. ^9^Center for Precision Health, McWilliams School of Biomedical Informatics, The University of Texas Health Science Center at Houston, Houston, TX 77030, USA. ^10^Department of Pediatric Hematology & Oncology, University Hospitals Cleveland Medical Center, Cleveland, OH, 44106, USA.

**Supplementary Figure Legends**

**Supplementary Fig.S1**. **oHSV therapy induces IGF2 gene expression in virus-infected tumor cells.** (**A**) Volcano plots of mRNA-Seq presented in Figure 1A, which identified 7,078 and 6,843 differentially expressed genes (DEGs) that were upregulated and 13,916 and 10,980 DEGs downregulated following rHSVQ infection in GBM12 and MDA468 respectively (n = 4/group). (**B**) IGF2 induction after G207 treatment in patients with recurrent GBM did not reach significance but had a notably limited sample size (n = 3). (**C**) qRT-qPCR of murine glioma (005) cells infected with or without a dose escalation of rHSVQ (i.e., 0, 0.1, 0.5, and 1) for 6 and 16 hours and GBM12 cells infected with or without rHSVQ (MOI = 0.1) for 0.5, 1, 3, and 6 hours revealed that oHSV therapy induces IGF2 gene expression in a time- and dose-dependent manner. IGF2 expression levels were normalized using GAPDH expression and presented as a fold change relative to uninfected cells (n = 3/group). (**D**) Released IGF1 level from culture media (CM) of numerous primary GBM cells and U251T3 glioma cell line infected with or without rHSVQ (MOI = 1, 24h). (**E**) Kaplan-Meier survival curves of all glioma patients tested in the Chinese Glioma Genome Atlas (CGGA, n = 983), reveal a significant survival benefit of reduced IGF2 expression but not IGF1 expression. Patients are stratified using a median split into “high” or “low” gene expression designated as above or below the median. Log-rank and Wilcoxon p values are labelled on top of each survival curve. All data are presented as means ± SEM with *p<0.05, **p<0.01 unless otherwise specified.

**Supplementary. Fig. S2.** **oHSV therapy induces IGF2 gene expression in an NFκB-dependent manner.** (**A**) The role for IGF2P4 was explored in vivo as described in Figure 2C by implanting GBM12-IGF2P4-Luc intracranially then treating intratumorally with PBS or rHSVQ (5 × 10^5^ pfu). IGF2 promotor activation was measured by in vivo bioluminescence imaging eight hours before and after viral treatment, revealing no significant change in IGF2P4 promoter activity upon viral injection compared to sham PBS injection (n = 6/group). (**B**) GSEA plots of NFκB signaling in mRNA-Seq performed on GBM12 and MDA468 cells (Fig. 1) reveals a significant enrichment of STAT3 and GATA2 gene expression upon rHSVQ infection. (**C**) KEGG pathway analysis depicting the top 10 upregulated pathways in rHSVQ-infected MDA468 cells (mRNA-Seq performed in Fig.1) demonstrates a similar pattern of upregulation of the NFkB signaling pathways seen in GBM12 cells (Fig. 2E). (**D**) Analysis of all glioma patients samples in the CGGA (n = 983) reveals a significant correlation between IGF2 and STAT3 gene expression (left) and between IGF2 and GATA2 gene expression (right), providing external validity for these as genetic regulators of IGF2 expression. Log2-transformed mRNA expression data were obtained. Linear regression estimates are shown as a trend line. (**E**) qRT-PCR of GATA2 (left) and IGF2 expression (right) with GATA2 knockdown demonstrates that rHSVQ infection is neither necessary nor sufficient to induce IGF2 expression. RT-qPCR expression levels were normalized using 18S rRNA expression and presented as the fold change compared to uninfected cells. (**F**) rHSVQ infection does not activate STAT3 promoter. The primary GBM12 and GBM28 cells co-transfected with a firefly-luciferase reporter harboring a putative Stat3 DNA-binding site (pGL3-STAT3-fLuc) and pGL3-TK-Renila luciferase (pGL3-TK-rLuc) were infected with or without rHSVQ (MOI=0.1) and luciferase activity was analyzed 24 hours post virus infection, revealing no significant activation of STAT3 promoter upon viral treatment (n = 3/group). All Firefly Luciferase Activity assays were normalized as a ratio of Firefly/Renilla Luciferase activity and expressed as the fold change compared to uninfected controls. All data are presented as means ± SEM with *p<0.05, **p<0.01 unless otherwise specified.

**Supplementary. Fig. S3. The effect of specific inhibition of IGF2 on oHSV therapy.** (**A**) Kaplan-Meier survival curve of mice with intracranial 005 murine gliomas or DB7 murine BCBM tumors treated intra-tumorally with PBS or rHSVQ (5 x 10^5^ pfu) nine days after tumor implantation. Starting 24 hours post viral injection, mice were treated with 20 µg/mouse of isotype control IgG or an anti-IGF2 antibody 2 times a week for the duration of the study by intraperitoneal injection. Kaplan-Meir survival curves revealed that systemic treatment of anti-IGF2 antibody does not enhance therapeutic efficacy of oHSVs in intracranial glioma and BCBM tumor-bearing mice in vivo, possibly due to poor tissue penetration. (**B**) Using the same CM from Q- and D11mt-infected BC and GBM cell lines and primary GBM demonstrates specific binding affinity of IGF2RD11mt to human IGF1 (hIGF1, top) or murine IGF1 (mIGF1, bottom) was quantified by ELISA using a secondary HRP-conjugated anti-human IgGFc antibody. (**C-D**) An anti-IGF2 antibody treatment has no effect on viral replication. Primary GBM28 and MDA231 breast cancer cells were infected with rHSVQ (MOI = 0.05) then treated with 20 µg/ml of isotype control IgG or an anti-IGF2 antibody 1 hour post virus infection. Forty-eight hours post virus infection, cells and media were harvested, and virus titer was quantified using a standard plaque-forming assay which showed no significant difference in viral titer. (**C**). Representative fluorescence microscopy images of GFP-positive rHSVQ-infected GBM28 cells showing no difference in viral propagation between the two groups (**D**). All data are presented as means ± SEM with *p<0.05, **p<0.01 unless otherwise specified.

**Supplementary. Fig. S4. Local administration of an anti-IGF2 antibody significantly decreases oHSV therapy-induced Ly6G+ neutrophil/PMN-MDSC infiltration in DB7 murine BCBM tumor model.** Intracranial DB7 BCBM tumors were treated intratumorally with either isotype control IgG or an anti-IGF2 mAb 8 days after implantation (20 μg/mouse, n = 3/group). Two days following antibody treatment, mice were treated with PBS or rHSVQ (5 x 10^5^ pfu, 2d) and mice brain were harvested 48 hours post virus injection. Tumor sections were stained for Ly6G (red) and counterstained with 4’6-diamidino-2-phenylindole (DAPI), revealing a marked reduction in Ly6G+ infiltrating cells in mice treated with local IGF2 inhibition.

**Supplementary. Fig. S5. oHSV therapy increases Ly6G+ neutrophil/PMN-MDSC infiltration in orthotropic mouse models of brain and BCBM.** (**A**) Intracranial 005 murine glioma-bearing mice were treated intratumorally with PBS or rHSVQ (5 x 10^5^ pfu) 10 days post tumor implantation. Two days post virus injection, tumor-bearing brain hemispheres were analyzed for CD11b^high^/CD45+/Ly6G+ gMDSC and CD11b^high^/CD45+ monocyte derived macrophage infiltration and activation by flow cytometry. (**B**) Intracranial 005 murine glioma-bearing C57BL/6 mice were treated with PBS or rHSVQ (5 x 10^5^ pfu) with isotype control or anti-Ly6G+ neutrophils/PMN-MDSC depleting antibody same as Fig. 6H. All data are presented as means ± SEM with *p<0.05, **p<0.01 unless otherwise specified.

**Supplementary. Fig. S6. A single treatment of oHSV-D11mt does not significantly enhance survival in immunocompromised or immunocompetent orthotropic mouse models of GBM.** (**A**) Kaplan-Meier survival curves of GBM-bearing mice treated intra-tumorally with PBS, rHSVQ (Q), or oHSV-D11mt (D11mt) (5 x 10^5^ pfu) seven days after tumor implantation, revealing no significant benefit with a single injection. (**B**) Intracranial MDA231BRM human BCBM tumor-bearing athymic nu/nu mice were treated with PBS, rHSVQ, or oHSV-D11mt with isotype control or anti-Ly6G neutrophils/gMDSC depleting antibody same as Fig. 6H. (PBS+Isotype control IgG, n = 5; PBS+anti-Ly6G, n = 5; rHSVQ+anti-Ly6G, n = 10; oHSV-D11mt+anti-Ly6G, n = 10). All data are presented as means ± SEM with *p<0.05, **p<0.01 unless otherwise specified.

**Supplementary Fig. S1.**


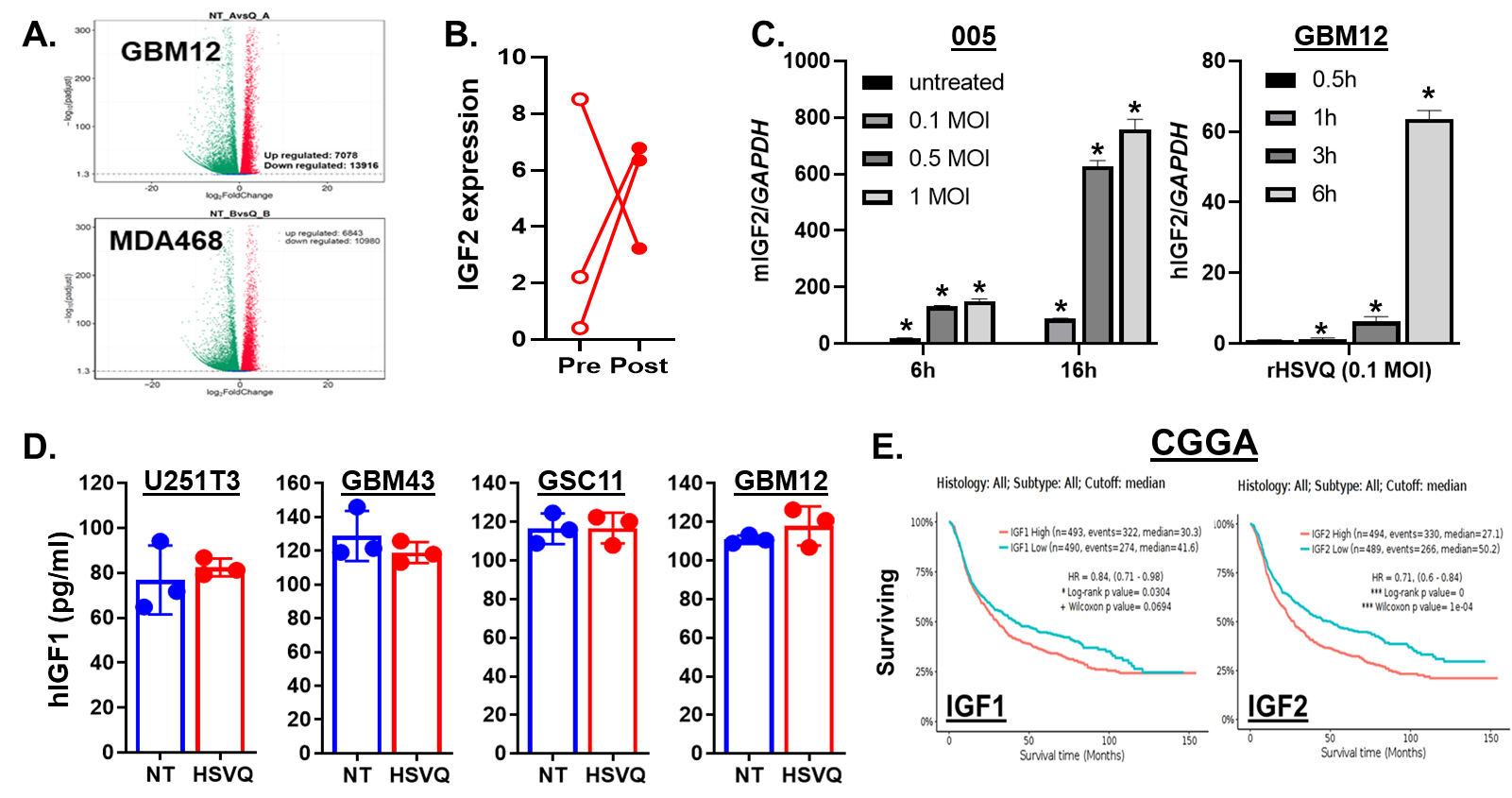


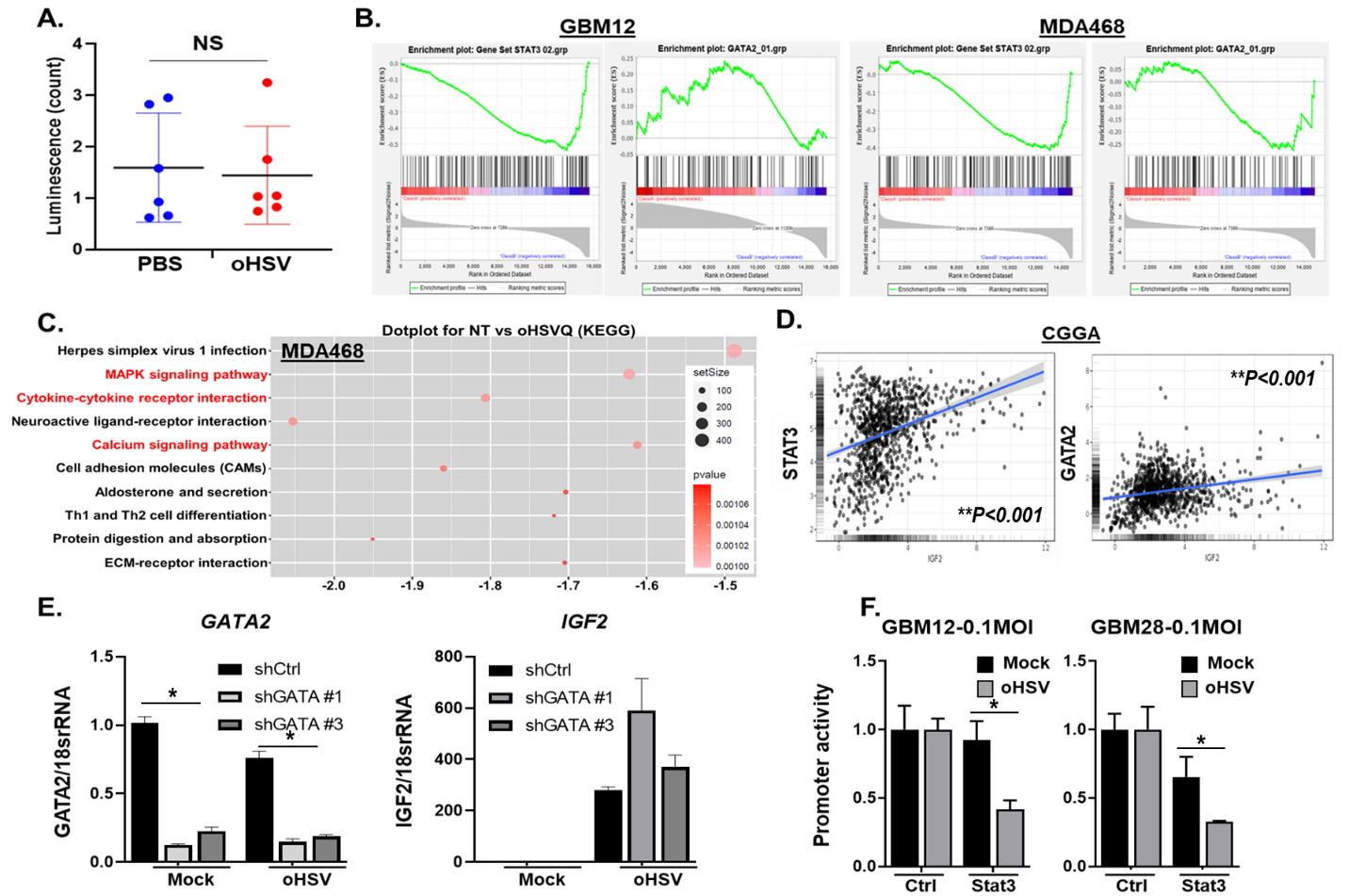
**Supplementary Fig. S2.**

**Supplementary Fig. S3.**


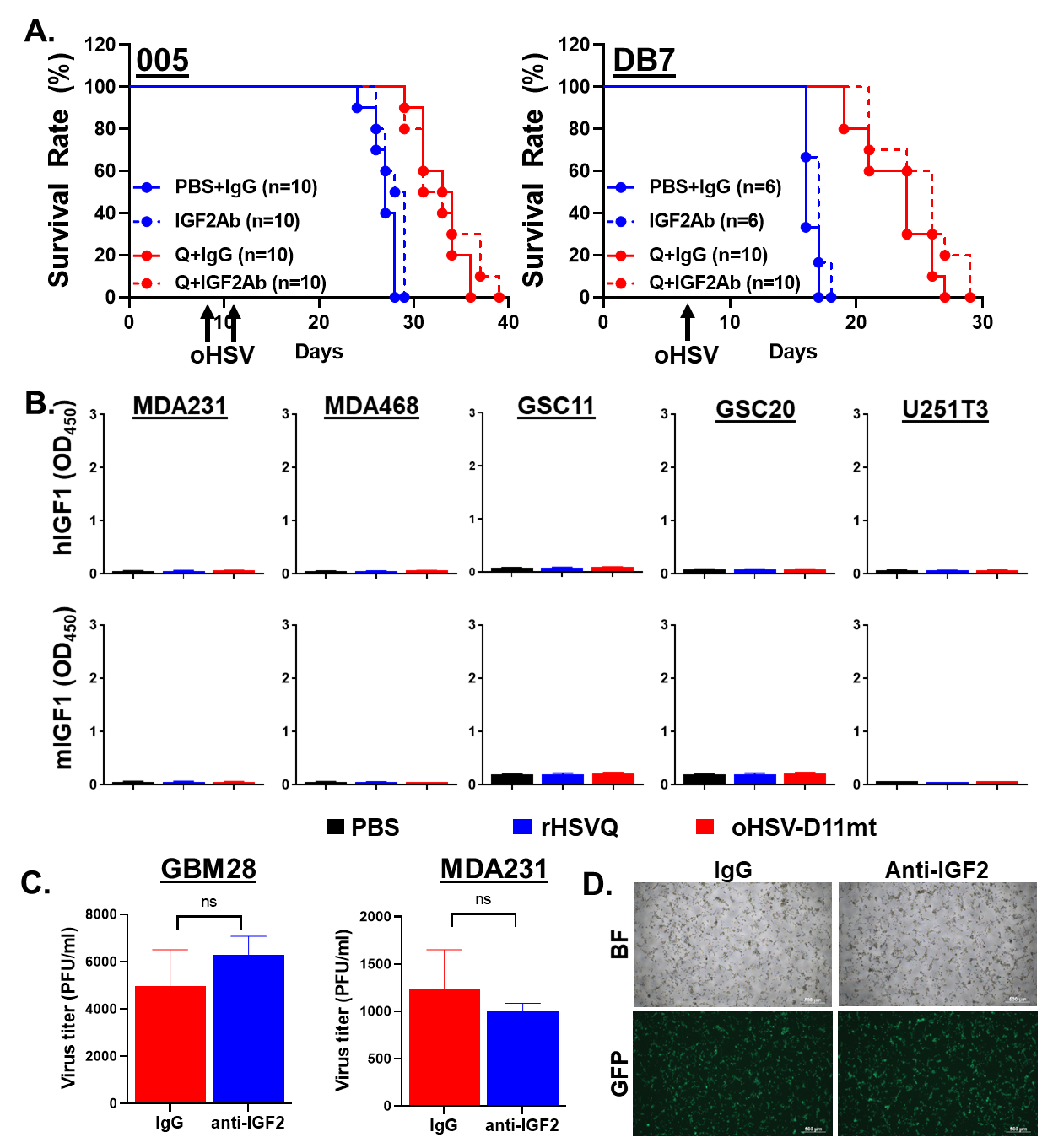


**Supplementary Fig. S4.**
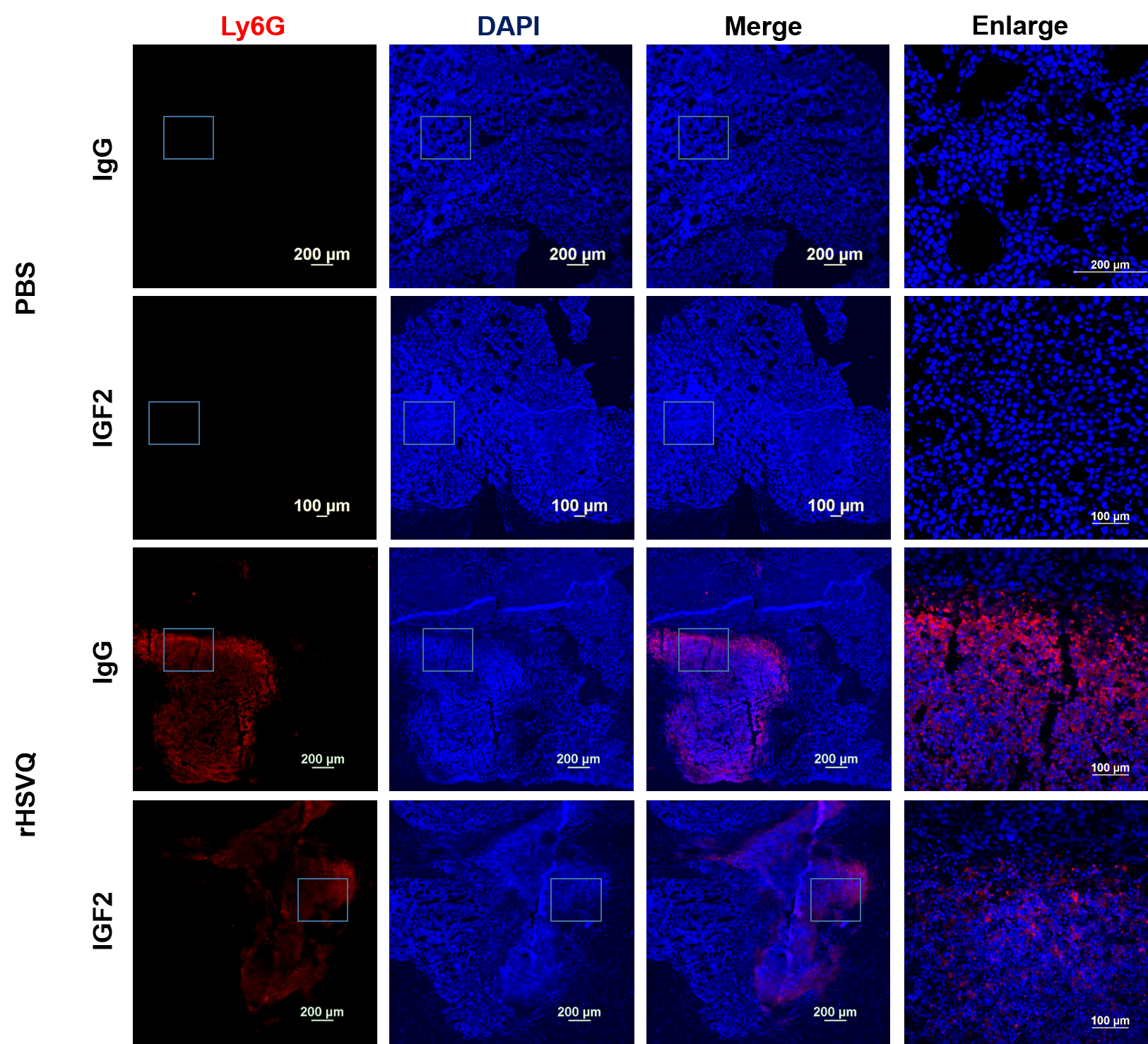


**Supplementary Fig. S5.**


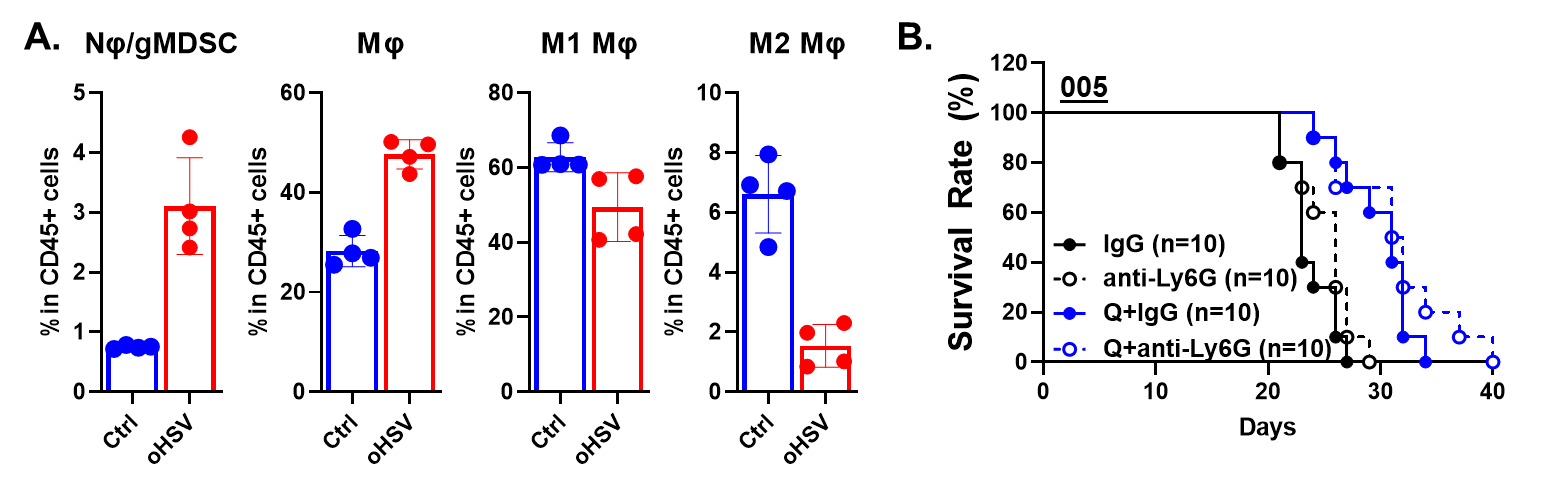


**Supplementary Fig. S6.**


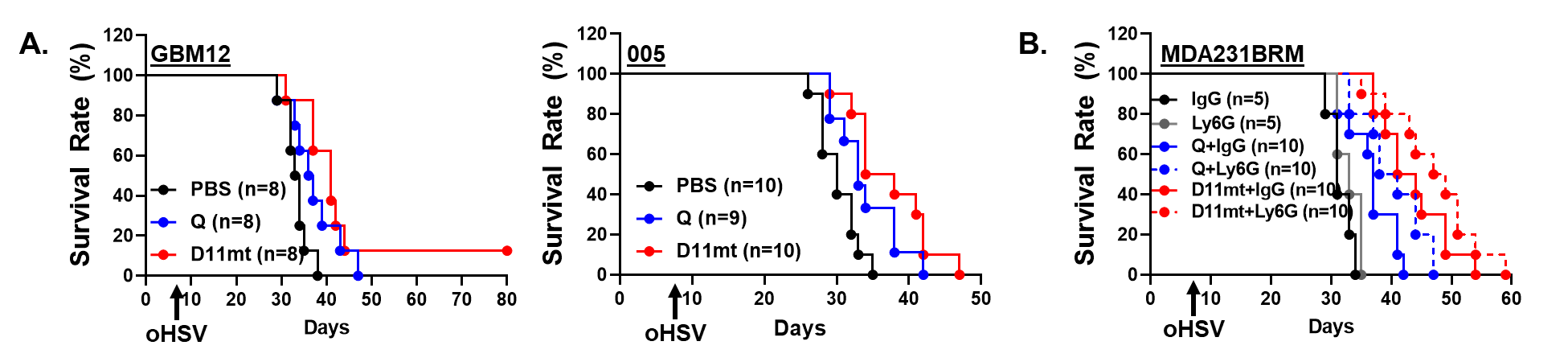


**Supplementary Materials and Methods**

*Cell lines and Oncolytic Herpes Simples Virus-1 (oHSV-1)*

Patient-derived primary GBM cells (i.e., GBM6, GBM12, GBM28, GBM39, and GBM43) were provided by Dr. Jann N. Sarkaria (Mayo Clinic, Rochester, MN) and GSC11 and GSC20 were obtained from MD Anderson Cancer Center (MDACC, Houston, TX). Murine 005 glioma cells were kindly donated by Dr. Tomotoshi Marumoto (The Salk Institute for Biological Studies, La Jolla, CA). Primary GBM cells were maintained as tumor spheres in neurobasal medium supplemented with 2% B27 without vitamin A, human EGF (20 ng/mL) (R&D Systems, Cat# 236-EG, Minneapolis, MN) and basic FGF (20 ng/mL) (R&D Systems, Cat# 3718-FB) in low-attachment cell culture flasks. DB7, murine breast cancer cells, and MDA-MB-231 and MDA-MB-468 human breast cancer cells were kindly donated by the lab of Dr. Michael Ostrowski (The Ohio State University, Columbus, OH) and maintained in Modified Eagle’s Medium (MEM; Gendepot, Houston, TX) supplemented with 10% fetal bovine serum (FBS). MDA-MB-231Br cells were kindly donated by Dr. Joan Massagué (Memorial Sloan Kettering Cancer Center, New York, NY). Vero cells derived from the kidney of an African green monkey and 4T1 murine breast cancer cells were obtained from the American Type Culture Collection (ATCC) and were maintained in Dulbecco’s Modified Eagle’s Medium (DMEM; Corning, Corning, NY) supplemented with 10% FBS. Primary GBM8, GBM12 (October 2018), GBM6, GBM28, GBM39, and GBM59 cells (December 2021) were authenticated by the Cytogenetics and Cell Authentication Core at MD Anderson Cancer Center via short tandem repeat (STR) profiling. U251T3 (January 2015) cells were authenticated by the University of Arizona Genetics Core. All cells are routinely monitored for changes in morphology and growth rate. All cells were maintained below passage 40 and are negative for *Mycoplasma*. All cells were incubated at 37°C in an atmosphere with 5% carbon dioxide, and maintained with 100 units/mL penicillin, and 0.1 mg/mL streptomycin. For our standard oncolytic HSV (oHSV), we used rHSVQ1, which includes a disruption in the UL39 locus and doubly deleted γ34.5 genes (1). We used previously described technology (2), to generate oHSV-D11mt (1). All viruses were propagated in Vero cells and their respective titers (plaque forming units per ml, PFU/ml) were quantified using the plaque forming unit assay in Vero cells as previously described (1).

*RNA Sequencing (RNA-Seq)*

RNA-seq was performed for GBM12 and MDA468 cells infected with or without 0.1 MOI of rHSVQ. Sixteen hours post viral infection, total RNAs were isolated using miRNeasy kit (Invitrogen, Carlsbad, CA) subjected to RNA isolation, and then submitted to Novogene (Sacramento, CA). Illumina sequencing was carried out at Novogene Bioinformatics Technology Co., Ltd., in Beijing, China. After the QC procedures, mRNA was enriched from total RNA using oligo(dT) beads. rRNA was removed using a specialized kit that leaves the mRNA. The mRNA was then fragmented randomly in fragmentation buffer, followed by cDNA synthesis using random hexamers and reverse transcriptase. After first-strand synthesis, a custom second-strand synthesis buffer (Illumina, Inc.) was added, with dNTPs, RNase H and Escherichia coli polymerase I to generate the second strand by nick-translation and, finally, AMPure XP beads were used to purify the cDNA. The final cDNA library is then ready after a round of purification, terminal repair, Atailing, ligation of sequencing adapters, size selection and PCR enrichment. Library concentration was first quantified using a Qubit 2.0 fluorometer (Life Technologies), and then diluted to 1 ng/gl before checking insert size on an Agilent 2100 and quantifying to greater accuracy by quantitative PCR (qPCR) (library activity >2 nM). Libraries were then fed into Novaseq6000 machines according to activity and expected data volume. A paired-end 150 bp sequencing strategy was used and all samples were sequenced to at least 6 Gp. To analyze the RNAseq of co-culture samples, we mapped the sequencing reads to the human and mouse genome references using STAR mapper to generate bam files. XenofilteR was further used for computational deconvolution of the mouse and human reads. The raw count of reads for the genomic features was evaluated by htseq-count. Differential gene expression was performed in edgeR package and Benjamini-Hochberg method was used for the multiple test adjustment. The genes with adjusted p-values less than 0.01 and absolute log_2_ (fold change) values greater than 1 were considered statistically significant.

*RNA-Seq Data Analysis*

Raw mRNA sequence reads were adaptor-trimmed and preprocessed with the Cutadapt (v1.15) software for quality control and data filtering, followed by alignment of clean RNA-seq reads of the human genome assembly GRCh38 with STAR (v2.5.3a). Uniquely mapped reads overlapping with target genes were counted by HTSeq-count with default parameter by using annotation from ENSEMBL v83. Only the genes with > 5 reads in at least one sample were included for differential expression analysis by DESeq2 software, which uses a model based on the negative binomial distribution. Resulting p-values were adjusted by using Benjamini and Hochberg’s approach to control for false discovery rate (FDR). The genes with fold change (FC) > 1.2 and FDR < 0.05 were considered as differentially expressed genes. Standard Gene Ontology (GO) and KEGG pathway-based enrichment analysis and Gene Set Enrichment Analysis (GSEA) of the IGF2-IGF1R signaling pathway were performed using RDAVID WebService (v1.19.0) and R package fgsea, respectively.

*RNA Isolation, cDNA Synthesis, and qRT-PCR*

We isolated total RNA using the RNeasy kit (Qiagen, Germantown, MD), and synthesized cDNA with the High-Capacity cDNA Reverse Transcription kit (Applied Biosystems, Waltham, MA). The qRT-PCR reactions were then performed with Fast SYBR Green Master Mix (Applied Biosystems). The qRT-PCR reactions were detected using an Applied Biosystems QuantStudio 3 thermocycler. As internal expression control, we used 18S rRNA or GAPDH expression for mRNA (designated in the relevant figure). Primers were designed with the Primer3 Plus software (https://primer3plus.com/) and were purchased from Integrated DNA Technologies (Coralville, Iowa). The primer sequences used are described in Supplementary Table S2.

*Dual Luciferase-NFκB Promoter Assay*

For the IGF2 promoter 3 and promoter 4 assay, a pGF-Luc vector was purchased from SBI (Palo Alto, CA) and IGF2 promoter 3 and promoter 4 were synthesized and subcloned in the pGF vector, generating pGF-IGF2p3-Luc and pGF-IGF2p4-Luc. The sequences and details are described in Supplementary Table S1. For the NFκB promoter assay, control- and NFκB response element (NRE)-Luciferase reporter system (pGL4.32 [luc2P/NF-κB-RE/Hygro] Vector), dominant negative IκBα-expressing plasmid (pGL34.2-dnIκBα), and pGL3-TK-Renila-Luc (normalizer) plasmids were purchased from Promega (Promega, WI, USA). The plasmid DNA was amplified from *E.coli*, then purified using the DNA Maxi kit (Qiagen, Germantown, MD, USA). GBM cells plated in 24-well plates one day prior was co-transfected with 200 ng of control-Luc or NRE-Luc and pGL3-TK-Renila-Luciferase, then with/without pGL34.2-dnIκBα DNAs in Lipofectamine 3000 per the manufacturer’s protocol (Invitrogen, Thermo Fisher, USA). Twenty-four hours post-transfection, cells were infected with rHSVQ (0.01 ~ 0.1 MOI). Twnety-four hours post co-transfection with control-Luc or NRE-Luc and pGL3-TK-Renila-Luciferase, cells were infected with or without rHSVQ (0.01 ~ 0.1 MOI). One hour post virus infection, cells were treated with DMSO or 5 uM of Bay11-7082 (Millipore Sigma, Cat#B5556, St. Louis, MO) for 8 hours. The cells were then lysed with Passive Lysis Buffer (Promega, Madison, WI, USA) and assayed with Dual Luciferase Assay kit (Promega, Madison, WI, USA) according to the manufacturer’s instruction.

*Chromatin Immunoprecipitation (ChIP) Assays*

Cells infected with/without rHSVQ (0.1 MOI) for 16 hours were cross-linked in 1% formaldehyde with continuous rotation at room temperature for 10 mins and quenched with 125 mmol/l of glycine for 5 mins. After two times of washing with PBS, cell lysates were sonicated, followed by chromatin shearing to a target peak size of 300~ 600 bp size (Covais). Fragmented chromatins were added into the ChIP dilution buffer [16.7 mM Tris–HCl (pH8.1), 167 mM NaCl, 1.2 mM EDTA, 1.1% Triton X-100, 0.01% SDS, and inhibitor cocktail]. Samples were then incubated with an NFκB antibody (Cat# 8242, Cell signaling, US) at 4 °C. Normal rabbit IgG was used as a negative isotype control. Immune complexes were precipitated using Protein A/G resin. After elution and reverse crosslinking at 65 °C, fragmented DNA was purified with a mini-column. Precipitated chromatins were then used as the template for qPCR. Based on TRANSFAC analysis (http://genexplain.com/transfac/), we identified two NFκB binding sites on the promoter 3 (IG2P3) and promoter 4 (IGF2P4) of IGF2. qPCR was carried out using following primer pairs. NFκB binding site in IGF2P3 (Forward 5'-GCAACAACCAGCAAGGAC-3' and Reverse 5'- CACCAGGAGGCTGCACTG-3'); NFκB binding site in IGF2 promoter 4 (Forward 5'- GGAGGAGAACCCACAACTCGG-3' and Reverse 5'- CCCCACCTTCTCCTCCAC-3').

*Quantification of human and murine IGF2 ELISA*

Human and murine IGF2 protein concentration was quantified in the culture media (CM) of treated cells using the human IGF2 and murine IGF2 DuoSet ELISA kit (R&D Systems, Cat# DY410, Minneapolis, MN) according to the manufacturer's recommendations. Serial dilutions of a known concentration of purified recombinant human or murine IGF2 were used to establish a standard curve for quantification.

*Cell Proliferation Assay*

Primary GBM cells were plated in 96-well plates containing 10000 cells/well in 50uL according to their standard culture conditions stated above. Seeded cells were infected with rHSVQ at 0.01, 0.05, and 0.1 MOI in a final volume of 25uL of 2% FBS-containing media and one hour later treated with 20 nM of a human (3 AF-292-NA, R&D Systems) or mouse IGF2 (3 AF792, R&D Systems) neutralizing antibody. Cells were incubated for 72 hours at 37℃ and cell viability was measured using a cell proliferation kit (Roche) following manufacturer’s protocol. Absorbance was measured using a Synergy HT Multi-Mode Microplate reader (Biotek), and cell viability was normalized to uninfected control cells and data were presented as percentage of cell viability.

*Binding affinity test by ELISA*

MaxiSorp 96-multiwell plates (Corning) were coated with 100 ng/ml of recombinant human or mouse IGF1 or IGF2 (R&D Systems) dissolved in 0.05 mol/L carbonate/bicarbonate buffer (pH 9.6) and incubated overnight at 4°C. The culture media (CM) collected from the PBS-, rHSVQ-, or oHSV-D11mt-treated cells was incubated for 2 hours at room temperature. The binding of the D11mt to IGF1 or IGF2 was assayed, using a goat anti-human IgG-Fc-HRP antibody (Jackson ImmunoResearch Laboratories, West Grove, PA).

*Co-culture Assay with peripheral blood mononuclear cells (PBMCs)*

Human PBMCs were isolated by density centrifugation with Ficoll-Plaque Cat# 45-001-751 Fisher Scientific, Waltham, MA) from healthy donors’ buffy coats (Gulf Coast Regional Blood Center, Houston, Texas, USA). To analyze immune cell-mediated tumor cell killing *in vitro*, rHSVQ- or oHSV-D11mt-infected glioma and BC cells were co-cultured with human PBMC (at 5:1 ratio of PBMC to cancer cells). Five days after co-culture, culture media (CM) and cells were collected and cells were stained with a CD45 antibody and live/dead fixable aqua cell stain and then analyzed by flow cytometry. CMs were analyzed for human IFNγ secretion using ELISA.

*Animal Studies*

All mouse housing and experiments were performed in accordance with the Animal Welfare Committee at the University of Texas Health Science Center in Houston guidelines and have been approved by the Institutional Review Board. Six- to eight-week-old outbred male and female athymic nu/nu and Balb/c were purchased from Jackson Laboratory. NSG, Balb/c nu/nu, and Fvb/n mice were obtained from Jackson Laboratory and bred in our facility at the University of Texas Health Science Center in Houston.

For intracranial tumor studies, anesthetized mice were fixed in a stereotactic apparatus and a burr hole was drilled in the right hemisphere at 2 mm lateral and 1 mm anterior to the bregma, to a depth of 3.5 mm. 1 x 10^5^ GBM12 and GBM28 human primary GBM cells, and murine glioma cells, DB7 and 4T1 murine breast cancer cells were implanted intracranially. Seven days post tumor cell implantation, mice were randomized and injected intratumorally with PBS or 5 x10^5^ pfu of rHSVQ or oHSV-D11mt. Animals were observed daily and euthanized at the indicated time points or when they showed signs of morbidity (i.e., biological endpoints including hunched posture and weight loss).In vivo Luciferase Imaging

Primary GBM12 cells stably expressing luciferase under IGF2 promoter 3 or 4 (GBM12-IGF2p3-Luc or GBN12-IGF2p3-Luc) were intracranially implanted into NSG mice as described above. Tumor-bearing mice were injected intratumorally with 5 × 10^5^ pfu of rHSVQ 21 days following tumor cell implantation. For in vivo studies, luciferase imaging was performed before and 8 hours post viral injection. Mice were injected with Luciferin solution (25mg/ml in PBS, dose of 100 mg/kg, Perkin Elmer, Waltham, MA) by intraperitoneal injection and anesthetized using isoflurane. The anesthetized mice were placed on non-fluorescent black paper on the imaging platform of an IVIS Lumina II to reduce background noise. Bioluminescence was detected by ZFOV-24 zoom lens-installed IVIS Lumina Series III Pre-clinical In Vivo Imaging System (Perkin Elmer, Waltham, MA). The luminescence intensity was expressed as averaged Radiance [p/s/cm²/sr].

*Processing of Tumor tissue for Flow Cytometry*

Six- to eight-week old female Fvb/n or Balb/c mice were obtained from Jackson Laboratory (Jackson laboratory, Bar Harbor, ME). 005 (1×10^5^ cells) were stereotactically implanted in the right hemisphere of mice as described above. 14 days post tumor implantation, mice were treated intratumorally with rHSVQ (5 x 10^5^ pfu) or PBS. Two and seven days after viral injection, tumor-bearing hemisphere were excised and digested using Tumor Dissociation Kit (Miltenyi Biotech) and the gentleMACS Octo Dissociator (Miltenyi Biotech). Cells were subsequently passed through a 70µm cell strainer and finally underwent debris removal using debris removal solution (Miltenyi Boitech) and red blood cell lysis by ACK buffer (Lonza, Basel, Switzerland) according to the manufacture’s instruction. Cells were then blocked with an anti-CD16/CD32 antibody (Invitrogen, RRID: AB_467133) and extracellular targets were stained at 4℃ for 30 minutes. For intracellular staining, cells were fixed and permeabilized using Cytofix/Cytoperm buffer (BD Biosciences, RRID: AB_2869008), then washed once in Perm/Wash buffer (RRID: AB_2869011) and incubated with antibodies for 30 minutes at 4℃. All antibodies used are listed in Supplementary Table S3. Stained cells were analyzed using a Cytoflex (Beckman Coulter, Brea, CA). Cell numbers were counted using CountingBright absolute counting beads per the manufacturer instruction (Invitrogen). Single stain controls for compensation for each fluorochrome were prepared using cells or compensation beads (Invitrogen, 01-222-42). Data was analyzed using FlowJo software.

*Immunofluorescence and Immunohistochemistry*

Mouse brains were fixed in 4% formaldehyde, followed by 30% sucrose at 4°C, and embedded in Paraffin. Representative coronal sections were stained with anti-IGF2 (3 MA5-17096, Invitrogen, Waltham, MA), anti-phospho-IGF1R (ab39398, Abcam, Cambridge, UK), anti-HSV-1 (ab9533, Abcam), and anti-Ly6G antibody (*ab25377, Abcam)*. SignalStain Boost IHC Detection Reagent and DAB substrate kit (Cell Signaling Technology) was used and then the sections were counterstained with DAPI or hematoxylin. Imaging was obtained with EVOS-FL Auto2 (Invitrogen) or NIKON A1R-MP (NIKON, Tokyo, Japan).

**References**

1. H. Wakimoto, G. Fulci, E. Tyminski, E. A. Chiocca, Altered expression of antiviral cytokine mRNAs associated with cyclophosphamide's enhancement of viral oncolysis. *Gene Ther* **11**, 214-223 (2004).

2. K. Terada, H. Wakimoto, E. Tyminski, E. A. Chiocca, Y. Saeki, Development of a rapid method to generate multiple oncolytic HSV vectors and their in vivo evaluation using syngeneic mouse tumor models. *Gene Ther* **13**, 705-714 (2006).

3. N. Svitek, I. Gerhauser, C. Goncalves, E. Grabski, M. Doring, U. Kalinke, D. E. Anderson, R. Cattaneo, V. von Messling, Morbillivirus control of the interferon response: relevance of STAT2 and mda5 but not STAT1 for canine distemper virus virulence in ferrets. J Virol **88**, 2941-2950 (2014).
